# Fecal Microbiota, Forage Nutrients, and Metabolic Responses of Horses Grazing Warm- and Cool-Season Grass Pastures

**DOI:** 10.3390/ani13050790

**Published:** 2023-02-22

**Authors:** Jennifer R. Weinert-Nelson, Amy S. Biddle, Harini Sampath, Carey A. Williams

**Affiliations:** 1 Department of Animal Sciences, Rutgers, The State University of New Jersey, New Brunswick, NJ 08901, USA; 2 Department of Animal and Food Sciences, University of Delaware, Newark, DE 19711, USA; 3 Department of Nutritional Sciences, Rutgers, The State University of New Jersey, New Brunswick, NJ 08901, USA; 4 Rutgers Center for Lipid Research, New Jersey Institute for Food, Nutrition, and Health, Rutgers, The State University of New Jersey, New Brunswick, NJ 08901, USA

**Keywords:** equine microbiome, glycemic response, non-structural carbohydrates, rotational grazing

## Abstract

**Simple Summary:**

Incorporating warm-season grasses into traditional cool-season grass equine rotational grazing systems can increase pasture availability during hot, dry months and bridge the “summer slump” forage gap. The objective of this study was to evaluate the impacts of this pasture management practice on the equine microbiome and to explore relationships between the fecal microbiota, forage nutrients, and metabolic responses of grazing horses. Results of this study indicate that distinct changes in microbial community structure and composition occur as horses adapt to different forages and that shifts in the microbial community were most influenced by forage non-structural carbohydrates and crude protein, rather than fiber. Interrelationships were found between these nutrients, glycemic responses, and *Akkermansia* and *Clostridium butyricum*. These bacteria were also found to be enriched in horses adapted to warm-season grasses. While the results of this study suggest that integrating warm-season grasses may not offer substantial metabolic benefits in healthy adult horses, this study did reveal new insights and targets for future research necessary to better understand the function of *Akkermansia* and *Clostridium butyricum* in the hindgut microbiome of grazing horses and possible roles in modulation of equine metabolic health.

**Abstract:**

Integrating warm-season grasses into cool-season equine grazing systems can increase pasture availability during summer months. The objective of this study was to evaluate effects of this management strategy on the fecal microbiome and relationships between fecal microbiota, forage nutrients, and metabolic responses of grazing horses. Fecal samples were collected from 8 mares after grazing cool-season pasture in spring, warm-season pasture in summer, and cool-season pasture in fall as well as after adaptation to standardized hay diets prior to spring grazing and at the end of the grazing season. Random forest classification was able to predict forage type based on microbial composition (accuracy: 0.90 ± 0.09); regression predicted forage crude protein (CP) and non-structural carbohydrate (NSC) concentrations (*p* < 0.0001). *Akkermansia* and *Clostridium butyricum* were enriched in horses grazing warm-season pasture and were positively correlated with CP and negatively with NSC; *Clostridum butyricum* was negatively correlated with peak plasma glucose concentrations following oral sugar tests (*p* ≤ 0.05). These results indicate that distinct shifts in the equine fecal microbiota occur in response different forages. Based on relationships identified between the microbiota, forage nutrients, and metabolic responses, further research should focus on the roles of *Akkermansia* spp. and *Clostridium butyricum* within the equine hindgut.

## 1. Introduction

Integrating warm-season grasses into traditional cool-season grass rotational grazing systems can provide productive pasture for grazing horses during the hot, dry months of the “summer slump” period [1,2]. Despite reported benefits for pasture yield [2,3] the impacts of this practice on equine metabolic health and the hindgut microbiome have not been previously investigated.

Warm- and cool-season grasses have different mechanisms for storage of soluble carbohydrates [4], with non-structural carbohydrate (NSC = sugars + starch + fructans) concentrations in cool-season grasses typically greater than that of warm-season grasses [1,5]. These differences in NSC content are of interest in equine management as current feeding recommendations for horses with existing metabolic dysfunction include limiting dietary NSC concentrations [6,7,8]. Thus, lower-NSC warm-season grasses have been suggested as an alternative forage source [1,9]. Feeding supplemental concentrate higher in NSC has been shown to lower insulin sensitivity in horses [10,11,12], but over the relatively smaller range of NSC concentrations observed in forages, potential benefits of limiting NSC intake are less clear [13,14,15]. However, glycemic and insulinemic responses of horses grazing low-NSC warm-season grasses have not been extensively evaluated.

In addition to the potential metabolic effects, transitioning horses between forage types may also have implications for equine gastrointestinal health. Diet has been identified as a dominant factor shaping the community structure of the gut microbiota both in humans and across animal species including horses [16,17]. However, prior studies on the influence of diet on the equine hindgut microbiome have focused primarily on concentrate vs. concentrate or concentrate vs. forage diets [18,19,20,21]. Recent studies have demonstrated that type of hay (alfalfa vs. grass) and transitions between hay and pasture grass can impact equine cecal or fecal microbial community composition [22,23]. Overall, few studies have evaluated the hindgut microbiome of grazing horses and only one previous study has been conducted in horses grazing cool- vs. warm-season pasture grasses [24].

A number of studies have begun to explore associations between the equine hindgut microbiota and metabolic health [25,26,27,28], but there is a lack of information on the interplay between forage nutrients, the hindgut microbiome, and metabolism of grazing horses. Biddle et al. [28] explored relationships between abundance profiles of fecal microbial taxa, feed types (pasture, hay, or hay supplemented with concentrate), and blood analytes including circulating glucose and insulin, finding negative correlations between insulin and over 50 taxa. Studies conducted in mouse models have conclusively demonstrated that changes in diet influence host metabolism in a microbiome-dependent manner [29,30]. Thus, there is a need for future research to better understand potential roles of the gut microbiota in modulating metabolism of grazing horses and the influence of specific forage nutrients on these interactions. While shifts in hindgut microbial communities have been documented in pastured horses over time, little is known regarding relationships between these changes in bacterial composition and forage nutrient profiles or metabolic responses of grazing horses. Therefore, the aims of this study were to characterize shifts in glucose metabolism and the fecal microbiota of horses adapted to different forage types and to explore relationships between forage nutrients, microbial composition, fecal metabolites, and metabolic responses of grazing horses.

## 2. Materials and Methods

Research was conducted in 2018 at the Ryders Lane Environmental Best Management Practices Demonstration Horse Farm (Rutgers, The State University of New Jersey; New Brunswick, New Jersey, NJ, USA). Weather data for the study period and historical averages are presented in Table S1 [31]. 

### 2.1. Grazing Systems

Two separate 1.5 ha integrated warm- and cool-season rotational grazing systems were utilized in this study. Grazing system design and management were as published in previous companion studies [2,24]. In brief, warm-season grass pasture sections contained either *Wrangler* bermudagrass [BER; *Cynodon dactylon* (L.) Pers.; Johnston Seed Company, Enid, OK, USA] or *Quick-N-Big* crabgrass [CRB; *Digitaria sanguinalis* (L.) Scop.; Dalrymple Farms, Thomas, OK, USA]. Mixed cool-season grass sections contained *Inavale* orchardgrass [*Dactylis glomerata* (L.)], *Tower* tall fescue (endophyte-free) [*Lolium arundinaceum* (Schreb.) Darbysh.], and *Argyle* Kentucky bluegrass [*Poa pratensis* (L.)] (DLF Pickseed, Halsey, OR, USA). Grazing management was guided by established best management practices [32].

### 2.2. Animal and Grazing Management

Use of animals in this study was approved by the Rutgers University Institutional Animal Care and Use Committee protocol #PROTO201800013. Eight adult Standardbred mares (age: 18 ± 0.71 yr; body weight (BW): 537 ± 17 kg; body condition score (BCS): 5–7)) were used in this study, with horses grouped by BW and BCS (Henneke Body Condition Score scale [33]. Horses were then randomly assigned to each system (*n* = 4 horses system^−1^). Prior to the study, oral sugar tests (OST) were administered to screen horses for impaired insulin sensitivity [34]. Insulinemic responses of all horses were found to be normal (peak insulin ≤45 mIU/L) [7]. Animal care and management have been previously detailed in companion studies [1,24]. Horse condition measures over the course of the study can be found in Table S2.

### 2.3. Fecal and Blood Sample Collection

Manual grab fecal samples were collected (0800 h) rectally from horses after 21 d adaptation to the initial hay diet in the spring (HAY-SP), cool-season grass pasture in the spring (CSG-SP), warm-season grass (WSG)—either BER or CRB during the “summer slump” period, and again following a return to cool-season grass in the fall (CSG-FA) and a final cool-season grass hay at the end of the grazing season (HAY-FA). See Figure S1 for a diagram of experimental design and sampling protocol. Due to delayed establishment and grazing of BER, only 17 days of grazing were possible prior to sample collection. Upon collection, samples were immediately placed on ice, transported to the laboratory, and then stored at −80 °C.

To determine impact of forage type within integrated systems on glycemic and insulinemic responses of grazing horses, OST [34] were conducted following adaptation to CSG-SP, WSG, and HAY-FA. On the evening prior to each collection period, horses were confined to dry lots at 2000 h with no access to either hay or pasture. Following the overnight fast, horses were moved into the barn facility at 0800 h. Two consecutive baseline blood samples were collected (15 min apart) by venipuncture at 0800 and an oral dose of Karo Syrup (0.25 mL/kg BW; modified dosing as utilized by Jacob et al. [12]) was immediately administered after the second baseline sample. Subsequent blood samples were collected at 30, 60, 90, 120, 180, and 240 min following Karo Syrup administration. Whole blood was collected into sodium heparin Vacutainer tubes, which were inverted several times before being placed on ice. Due to logistics of distance and travel time (~10 min from the barn to the laboratory), samples were transported to the laboratory after the 120, 180, and 240 min samples. In the laboratory, whole blood was centrifuged at 3700 rpm for 7 min. Following centrifugation, plasma was harvested from the Vacutainer tubes and aliquoted into microcentrifuge tubes, which were then stored at −80 °C.

### 2.4. Forage Sampling

Representative hand-clipped forage samples were also collected (0800–1000 h) on three days per period for analysis of nutrient composition. Pasture samples were collected according to previously published procedures [1,24,32] and dried (60 °C; 36 h minimum) in a Thelco oven (Precision Scientific, Chicago, IL, USA). Samples were ground (1 mm) and submitted to a commercial laboratory (Equi-Analytical Laboratories, Ithaca, NY, USA) for analysis by near-infrared spectroscopy. The mean nutrient composition of hay diets and pasture forages are shown in Table 1.

### 2.5. Analysis of Plasma Glucose and Insulin

Plasma glucose was analyzed by colorimetric assay (Glucose C-2, Wako Chemicals, Richmond, VA, USA), with the commercial kit adapted for microplate assay following manufacturer instructions. Plasma insulin was evaluated using an enzyme-linked immunoassay (Mercodia Equine Insulin ELISA, Mercodia, Winston-Salem, NC, USA) previously validated in horses [35]. Inter-assay and intra-assay coefficients of variation for glucose were 4.0% and 2.9%, respectively. Inter-assay and intra-assay coefficients of variation for insulin were 7.8% and 3.4%, respectively.

### 2.6. Fecal Sample Analyses

Fecal pH was measured in duplicate with a handheld Accumet pH meter (Fisher Scientific; Waltham, MA, USA) using a previously published protocol for preparation of fecal slurries [24,36]. Short-chain and branched-chain fatty acid (SCFA and BCFA, respectively) concentrations were determined by GC-MS analysis of fecal samples. The SCFA analyzed included acetate, propionate, butyrate, and valerate. The BCFA analyzed included isobutyrate, isovalerate and isocaproate. Sample preparation was performed according to a previously published protocol [37]. In brief, frozen fecal samples were weighed and deposited in bead tubes over dry ice. Feces were then resuspended in 1 mL of 0.5% phosphoric acid per 0.1 g of sample and tubes were beaten for 5 min at 22.5 rpm in a cold case. Samples were again frozen at −80 °C until analyzed by Gas Chromatography and Mass Spectrometry system GC-MS (Agilent Technologies, Santa Clara, CA, USA). Prior to GC-MS analyses, thawed fecal suspensions were re-homogenized and centrifuged (10 min at 17,949× *g*), with the aqueous phase extracted using diethyl ether in a 1:1 volume to volume ratio. Before analysis, 2-methyl hexanoic acid (Thermo Fisher Scientific, Dallas, TX, USA) was added to the organic phase extract as an internal standard. Samples were analyzed in duplicate, with independent extractions for each replicate. The specifications of the GC-MS system and analyses as well as data acquisition and procedures for SCFA/BCFA quantitation have been previously described by Honarbakhsh et al. [37] and Garcia-Villalba et al. [38].

Quick-DNA Fecal/Soil Microbe Kits (Zymo Research; Irvine, CA, USA) were used for DNA extraction (in triplicate). The highest yielding replicate (quantified with a Qubit 2.0 Flourometer [Invitrogen; Carlsbad, CA, USA]) was submitted to a commercial laboratory (RTL Genomics; Lubbock, TX, USA). Amplification of the V4-V5 region of the 16S rRNA gene was conducted using region specific primers (515F/926R) [39]; sequencing was conducted by Illumina MiSeq.

### 2.7. Sequence and Statistical Analysis

Sequence and statistical analyses were performed in QIIME 2 (Quantitative Insights into Microbial Ecology, v. 2020.8) [40] and R (v. 4.0.2) [41]. Network mapping was conducted in Cytoscape (v. 3.8.0) [42]. Animal was considered the experimental unit. 

Quality and chimera filtering of forward reads was conducted using DADA2 (read length = 185) [40,43,44]. Mafft and FastTree (q2-phylogeny plugin) were used to create trees for diversity analyses [45,46,47]. The lower quartile of Amplicon Sequence Variants (ASV) based on absolute abundance were removed (minimum frequency = 16; minimum samples = 4). The feature table was rarefied to a minimum sampling depth of 10,600 prior to α- and β-diversity analyses. The α-diversity metrics were analyzed by Kruskal–Wallis tests [48,49,50,51,52]. The β-diversity metrics analyzed included Weighted and Unweighted UniFrac by permutational ANOVA (PERMANOVA) [53,54,55,56,57,58,59]. Benjamini and Hochberg FDR adjustments for multiple pairwise comparisons were applied for all diversity analyses. Permutational multivariate analysis of dispersion (PERMDISP) was used to test homogeneity of dispersion [60]. 

To further explore differential abundances, ASV were then grouped into bacterial co-abundance groups (BCG) based on abundance profiles using Sparce Cooccurrence Network Investigation for Compositional Data (SCNIC) [61,62]. A random forest classifier with nested cross validation was applied to determine if forage type could be predicted based on BCG composition [63,64]. Features (BCG) were removed from the model based on importance scores generated by the random forest classifier in an iterative process (serial reduction with increments of 5) until the point at which model accuracy began to decline [65]. Relative abundances of remaining BCG and any uncorrelated ASV retained in the model following feature reduction processes were then analyzed by linear discriminant analysis effect size (LEfSe) to identify BCG specific to each forage type, with significance set at an LDA score >2.0 [66]. Taxonomy was then assigned using the latest SILVA database (SSU 138) [63,67,68,69,70].

Glucose and insulin response variables as well as SCFA/BCFA concentrations and fecal pH were analyzed by mixed model ANOVA in R, with grazing system, forage type and their interactions set as fixed factors and horse as the random factor in the initial model. The area under the curve (AUC) was calculated based on the trapezoid rule as the positive incremental AUC utilizing a published macro [71] in SAS (v.9.4 SAS Institute, Cary, NC, USA). Proxies for insulin sensitivity (fasting glucose-to-insulin ratio (FGIR); reciprocal of the square root of insulin (RISQI)) and insulin secretory response (modified insulin-to-glucose ratio (MIRG)) were calculated using baseline (fasting) values as previously described [72,73]. 

The relationship between forage nutrients and SCFA/BCFA fermentation metabolites (as well as pH) and fecal microbial community composition was then explored using random forest regression with nested cross validation to determine if nutrient concentrations and/or metabolite concentrations could be predicted based on bacterial abundance profiles. Spearman correlations between BCG and forage nutrients and fecal metabolites were analyzed in R. Relationships between forage nutrients/fermentation metabolites and BCG were then visualized in Cytoscape. Spearman correlations between metabolic variables and the BCG, as well as between forage nutrients and metabolic responses were also evaluated in R. 

For all variables analyzed by mixed model, model residuals were analyzed for normality using the Shapiro–Wilk test. Log, square root, or inverse data transformations were applied where appropriate for non-normal data. Means were separated using Tukey’s method. When analyzing pairwise comparisons of transformed data, means and standard errors were back-transformed to the original variable scale following application of Tukey’s method with the delta method. For all analyses which generated *p*-values, results were considered significant at *p* ≤ 0.05, with trends considered at *p* ≤ 0.10. Data for variables analyzed by mixed model are presented as means ± SEM. Overall analysis of microbiome, fecal metabolite, and glucose/insulin data did not reveal differences by grazing system. Therefore, grazing system was removed from models and results for combined data are presented with *n* = 8.

## 3. Results

### 3.1. Initial 16s rRNA Sequence Analysis

A summary of 16S rRNA gene sequencing reads before and after quality and chimera filtering as well as after filtering of low abundance features in the 40 samples analyzed for this study is shown in Table 2. There were 1264 distinct ASV in the final dataset (taxonomy can be found in Table S3).

### 3.2. Diversity Analyses

**Figure 1:**
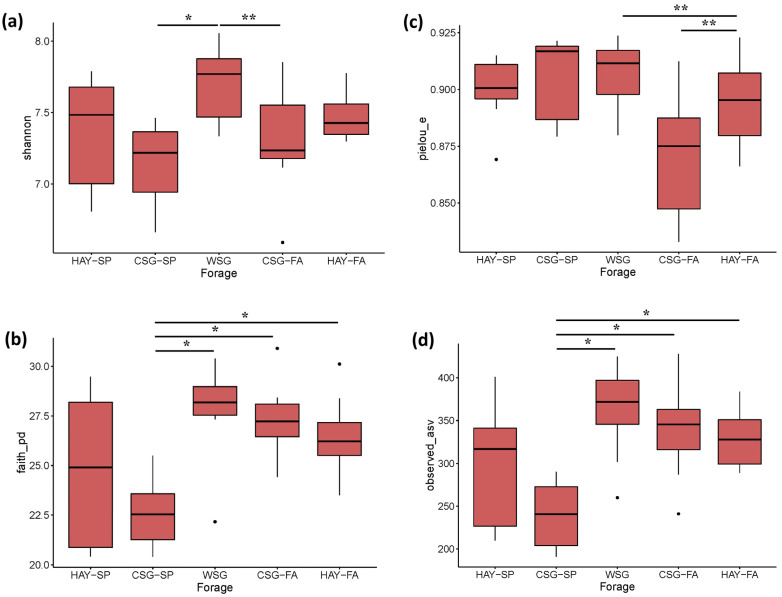
Fecal microbiota α-diversity across forages within an integrated rotational grazing system including: HAY-SP: initial standardized cool-season grass hay diet in the spring; CSG-SP: cool-season grass pasture in the spring; WSG: warm-season grass, either crabgrass or bermudagrass, in the summer slump period; CSG-FA: cool-season pasture in the fall; HAY-FA: final standardized cool-season grass hay diet in the fall. Shannon Diversity Index (**a**), Faith’s Phylogenetic Diversity (**b**), Pielou’s Evenness (**c**) and Observed ASV (**d**) were analyzed by Kruskal–Wallis tests with Benjamini and Hochberg FDR adjustments for multiple pairwise comparisons. A “⋅” indicates a statistical outlier in the data. A single asterisk indicates differences between forages at *p* ≤ 0.05. A double asterisk indicates a trend for differences between forages at *p* ≤ 0.10.

All α-diversity metrics evaluated, including the Shannon Diversity Index, Faith’s Phylogenetic Diversity, Pielou’s Evenness, and Observed ASVs, differed by forage (Kruskal–Wallis tests with Benjamini and Hochberg FDR adjustments; *p* < 0.03; Figure 1a–d). Shannon Diversity was greater when horses were adapted to WSG than CSG-SP (*p* < 0.05), and there was a trend for greater diversity when adapted to HAY-SP compared to CSG-SP (*p* < 0.08) and WSG vs. CSG-FA (*p* = 0.09). However, the only differences in evenness (Pielou’s Evenness) were trends for greater evenness in WSG and CSG-SP than in CSG-FA (*p* < 0.06). Conversely, richness (Observed ASVs) was greater in horses adapted to WSG, CSG-FA, and HAY-FA than CSG-SP (*p* < 0.02), but did not differ between horses adapted to HAY-SP and CSG-SP. Similarly, phylogenetic differences (Faith’s Phylogenetic Diversity) were also found between CSG-SP and subsequent forages (WSG, CSG-FA, HAY-FA; *p* < 0.02). 

**Figure 2:**
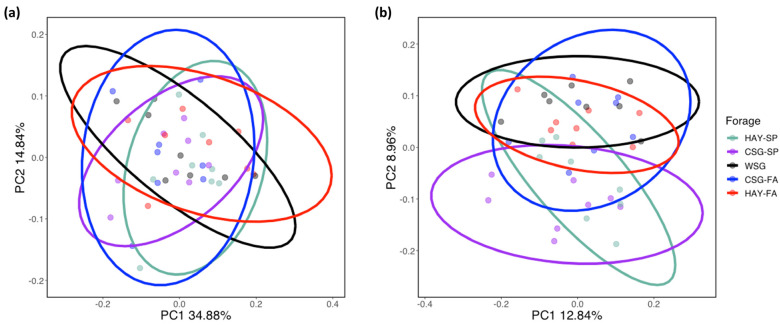
Fecal microbiota β-diversity across forages within an integrated rotational grazing system including: HAY-SP: initial standardized cool-season grass hay diet in the spring; CSG-SP: cool-season grass pasture in the spring; WSG: warm-season grass, either crabgrass or bermudagrass, in the summer slump period; CSG-FA: cool-season pasture in the fall; HAY-FA: final standardized cool-season grass hay diet in the fall. Weighted UniFrac (**a**) and Unweighted UniFrac (**b**) differed by forage based on analysis by Permutational ANOVA (PERMANOVA) (*p* ≤ 0.05).

Principal coordinate analysis of β-diversity metrics including Weighted and Unweighted UniFrac did not reveal distinct clustering by forage (Figure 2a,b). However, statistical analysis by PERMANOVA (with Benjamini and Hochberg FDR adjustments) found significant differences in these measures (*p* ≤ 0.02). Subsequent PERMDISP analysis confirmed that these differences were not due to differences of variance or dispersion within groups. Unweighted UniFrac differed for all pairwise comparisons of forages (*p* < 0.02), with the exception of WSG vs. CSG-FA and WSG vs. HAY-FA for which there were trends for differences (*p* < 0.10). In contrast, there were no significant differences in any pairwise comparisons of forages for Weighted UniFrac. The percent of variation explained by PC1 was almost 3 times greater for Weighted than for Unweighted UniFrac, indicating an influence of abundance profiles in addition to phylogenetic differences. 

### 3.3. Differential Abundance

Application of SCNIC identified 333 BCG, with 224 individual ASV remaining ungrouped. Iterative reduction of features (BCG) based on random forest model importance scores revealed that model accuracy increased through reduction to the top 65 features (0.90 ± 0.09). Further feature reduction to the top 25 features (based on importance scores) did not impact random forest model accuracy (0.90 ± 0.09 with the top 25 features retained). All retained features were BCG; no ungrouped ASV remained in the reduced feature set. These 25 BCG were retained for further analysis, with 11.54% of the total microbial community abundance represented by the reduced 25 BCG feature set. The strength of the random forest model accuracy score indicated that forage type could be predicted based on bacterial composition, and that, conversely, bacterial community composition was influenced by forage. 

Subsequent Linear discriminant analysis Effect Size (LEfSe) analysis conducted on features retained from the random forest classification modelling identified 6 BCG as markers of HAY-SP, 3 BCG enriched in CSG-SP, 5 BCG for WSG, 5 BCG for CSG-FA, and 5 BCG for HAY-FA (LDA > 4.0; *p* < 0.03). Forage-specific BCG markers as well as taxonomic classifications of subsequent linear discriminant analysis Effect Size analysis conducted on features retained from the random forest classification modelling identified 6 BCG as markers of HAY-SP, 3 BCG enriched in CSG-SP, 5 BCG for WSG, 5 BCG for CSG-FA, and 5 BCG for HAY-FA (LDA > 4.0; *p* < 0.03). Forage-specific BCG markers as well as taxonomic classifications of individual ASV within each BCG are presented in Table 3, Table 4 and Table 5.

Numerous taxa were represented in BCG markers for multiple forages. At the family level, the BCG markers for HAY-SP contained twice as many ASV mapped to the *Lachnospiraceae* family as any other forage (CSG-SP: 0; WSG: 2; CSG-FA: 3; HAY-FA: 2 ASV). The family *Oscillospiraceae* also had ASV members of BCG markers for all forages but CSG-SP (HAY-SP: 3; WSG: 3; CSG-FA: 2; HAY-FA: 2 ASV). At the genus level, ASV assigned to *Christensenellaceae* R-7 group were present in BCG markers of both hay diets (HAY-SP: 2; HAY-FA:1 ASV) and WSG (2 ASV); ASV assigned to the *NK4A214 group* of *Oscillospiraceae* were among members of BCG identified as markers of HAY-SP (2 ASV), WSG (1 ASV), and CSG-FA (1 ASV). The BCG markers of both hay diets and CSG-FA each included an ASV mapped to *Rikenellaceae* RC9 gut group, and BCG markers of HAY-SP and WSG each contained ASV assigned to *Fibrobacter* and *Papillibacter*. The BCG markers of HAY-SP and CSG-FA contained ASV within *Catenisphaera* and *Lachnospiraceae* UCG-009. Amplicon sequence variants mapped to the genus *Clostridium sensu stricto* 1 were found in BCG markers of CSG-SP and WSG. The BCG markers of WSG and CSG-FA included ASV assigned to *Bacteriodales* RF16 group and the Family XIII AD3011 group of *Anaerovoracaceae*. The BCG markers of CSG-FA and HAY-FA each contained an ASV assigned to *Coprostanoligenes* group, *Lachnospiraceae* XPB1014 group and *Marvinbryantia*, and ASV assigned to Treponema were assigned to BCG markers of WSG and HAY-FA. 

Amplicon sequence variants assigned to *Ruminococcus*, *Pseudobutyvibrio*, *Anaerovibrio*, probable genus 10 of *Lachnospiraceae*, and the *p-251-o5* genus and family of the order *Bacteroidales* were only present in BCG markers identified for HAY-SP; no other ASV assigned to these taxa were found in BCG markers of other forages. An ASV within *Bacteroidales* BS11 gut group was the only taxa specific to BCG markers of CSG-SP. Amplicon sequence variants mapped to the genera *Akkermansia*, *Mogibacterium*, and the *Hallii* group of *Lachnospiraceae* as well as UCG-005 metagenome of *Oscillospiraceae* and *Clostridium butyricum* were only found in BCG markers of WSG. The BCG markers of CSG-FA contained ASV assigned to *Alloprevotella*, *Erysipelatoclostridium*, *Bacteroidales* UCG-001, the *WCHB1-41* genus, family and class within the class *Kiritimatiellae*, and *Denitrobacterium detoxificans*; these taxa were not identified in BCG markers of other forages. Taxa specific to only BCG markers of HAY-FA included ASV from the *Incertae Sedis* genus of *Ethanoligenenaceae* as well as *Streptococcus*.

### 3.4. Fecal pH and Fermentation Metabolites

**Figure 3:**
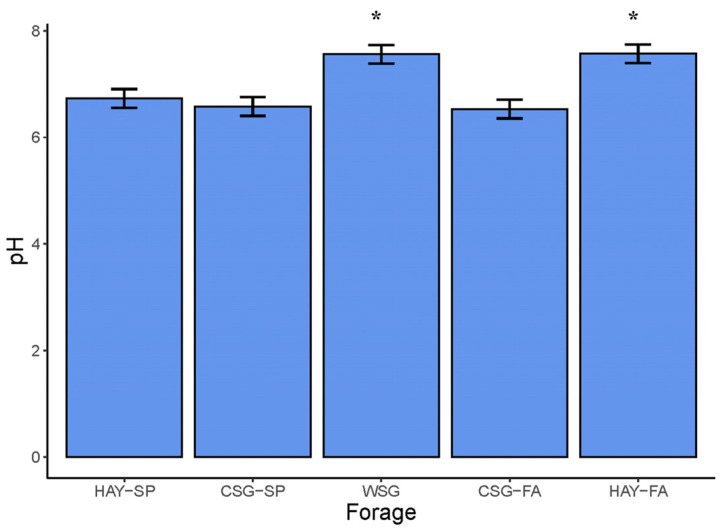
Fecal pH across forages within an integrated rotational grazing system including: HAY-SP: initial standardized cool-season grass hay diet in the spring; CSG-SP: cool-season grass pasture in the spring; WSG: warm-season grass, either crabgrass or bermudagrass, in the summer slump period; CSG-FA: cool-season pasture in the fall; HAY-FA: final standardized cool-season grass hay diet in the fall. Data are presented as least squares means ± SEM. A single asterisk indicates differences between forages at *p* ≤ 0.05 (mixed model ANOVA with Tukey’s post hoc adjustment).

Fecal pH differed by forage type (mixed model ANOVA with Tukey’s post hoc adjustment; *p* < 0.0001). Fecal pH was greater in horses adapted to WSG (7.56 ± 0.18) and HAY-FA (7.57 ± 0.18) than in HAY-SP (6.73 ± 0.18), CSG-SP (6.58 ± 0.18), or CSG-FA (6.53 ± 0.18; *p* ≤ 0.02; Figure 3). Fecal concentrations of short-chain fatty acids (SCFA) and branched-chain fatty acids (BCFA) also differed by forage (mixed model ANOVA with Tukey’s post hoc adjustment; *p* < 0.001; Table 6).

Unlike fecal pH, differences in SCFA and BCFA were primarily between pasture forages and the hay diets, with horses adapted to WSG often intermediate (numerically) between cool-season pasture and hay diets. Total BCFA were greater in CSG-SP, WSG, and CSG-FA than for either HAY-SP or HAY-FA (*p* < 0.05). Total SCFA were greater in CSG-SP and CSG-FA than HAY-SP or HAY-FA; WSG did not differ from CSG-SP and CSG-FA or HAY-SP, but was greater in comparison to HAY-FA (*p* < 0.002). Similarly, acetate was greater in CSG-FA than HAY-SP or HAY-FA, while WSG only differed from HAY-FA (*p* < 0.02). Acetate concentrations for CSG-FA also differed with HAY-FA (*p* = 0.0003), but there was only a trend for a difference between CSG-SP and HAY-SP (*p* = 0.09). Fecal butyrate was lower for HAY-FA than when horses were adapted to any of the pasture forages (*p* < 0.03), but there was no difference between HAY-SP and any other forage. Fecal propionate was lowest in horses adapted to HAY-FA (*p* < 0.002), but propionate concentrations were also lower for HAY-SP than either CSG-SP or CSG-FA (*p* < 0.03). Propionate did not differ between horses adapted to WSG and all other forages. Fecal valerate was lower for horses adapted to HAY-FA than CSG-SP or CSG-FA (*p* < 0.03) but did not differ from WSG or HAY-SP. Horses adapted to HAY-SP also had fecal valerate concentrations lower than CSG-SP or CSG-FA (*p* ≤ 0.0002). Isobutyrate was greater for all pasture forages in comparison to both HAY-SP and HAY-FA (*p* < 0.02). Isovalerate was greater in CSG-SP and CSG-FA vs. HAY-SP and HAY-FA (*p* ≤ 0.002), while concentrations in horses adapted to WSG were once again intermediate. Isocaproate was detected in all fecal samples, but concentrations were negligible and below the limit of quantification (<1.0 ug g feces^−1^).

### 3.5. Relationships between Fecal Microbiota and Metabolites

Random forest regressors were applied to determine if fecal pH and metabolite concentrations could be predicted based on microbial composition. Random forest regression did not support a strong influence of bacterial composition of the full microbial community on these fecal variables. Relatively weak predictive accuracy was found for isovalerate, valerate, hexanoate, and heptanoate (model R^2^ and *p*-values are shown in Table 7). Major SCFA including acetate, butyrate, and propionate could not be predicted based on BCG abundance profiles.

However, when random forest regressors were applied to only the top 25 BCG identified as most predictive of forage type, model accuracies improved for all fecal variables with the exception of valerate (Table 7). While predictive accuracy was still relatively weak, all metabolites (and pH) could be predicted with statistically significant accuracy (*p* ≤ 0.001).

**Figure 4:**
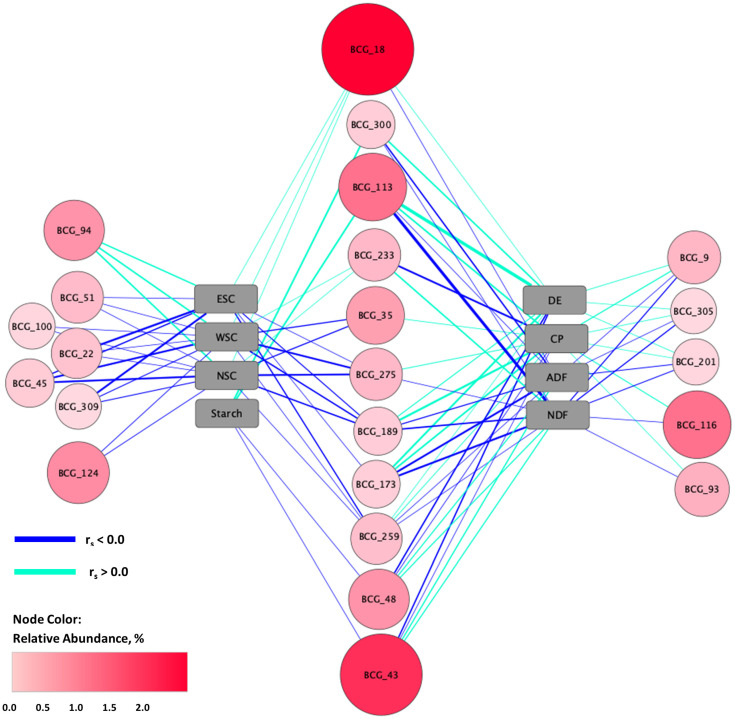
Network map of forage nutrients and bacterial co-abundance groups (BCG). Nutrients included digestible energy (DE), crude protein (CP) acid detergent fiber (ADF), neutral detergent fiber (NDF), non-structural carbohydrate (NSC), water-soluble carbohydrate (WSC) and ethanol-soluble carbohydrate (ESC). Correlations were evaluated between nutrient concentrations and relative abundance of BCG most predictive of forage type (based on random forest modeling of BCG composition). In addition to node color, node size was also mapped to BCG relative abundance. Line width is mapped to correlation strength, with wider lines indicating stronger correlations between nutrients and BCG. Significant Spearman correlations (r_s_ ≥ |0.30|; *p* ≤ 0.05) were included in the network map.

Individual correlations were found between 19 of these BCG and at least one fecal metabolite or pH (Spearman correlation; r_s_ ≥ |0.30|; *p* ≤ 0.05; Figure 4), with most BCG correlated with multiple fecal variables. There were positive correlations between BCG_300 and total SCFA and total BCFA as well as with acetate, butyrate, propionate, valerate, isobutyrate, and isovalerate, while this BCG was negatively correlated with hexanoate, heptanoate, and pH. The ASV members of this BCG were assigned to the genera *Lachnospiraceae XPB1014* and *Streptococcus*. There were also positive correlations between both BCG_9 and BCG_259 and total SCFA and BCFA in addition to acetate, butyrate, propionate, valerate, isobutyrate, and isovalerate; these BCG were negatively correlated with heptoanate. These BCG included multiple ASV assigned to *Lachnospiraceae* as well as ASV within the genera *Christensenellaceae R-7 group*, *Rikenellaceae RC9 gut group, Papillibacter*, and the *NK4214 group* of *Oscillospiraceae.* Positive correlations were also found between BCG_173 and BCG_201 and total SCFA and BCFA, acetate, propionate, valerate, isobutyrate, and isovalerate, with negative correlations between these BCG and both hexoanate and heptoanate. These BCG included ASV assigned to *Treponema*, *Rikenellaceae RC9 gut group*, the *NKA214 group* of *Oscillospiraceae*, and the *p-251-o5* and *F082* genera and families of *Bacteroidales*. Total SCFA and BCFA as well as acetate, butyrate, propionate, isobutyrate, and isovalerate were positively correlated with BCG_113, while hexanoate was negatively correlated. This BCG contained ASV assigned to taxa including *Synergistaceae*, *Bacteroidales RF16 group*, *Papillibacter*, *Christensenellaceae R-7 Group*, *Mogibacterium*, and *Clostridium butyricum*.

Seven BCG were positively correlated with total BCFA and some combination of propionate, valerate, isobutyrate, and isovalerate, while negative correlations were found between these BCG and hexanoate and/or heptanoate. These BCG included ASV members assigned to the family level for *Anaerovoracaceae*, *Oscillospiraceae*, and *Lachnospiraceae*. Other ASV within these BCG were assigned to *Lactobacillus equigenerosi* and the genera *Akkermansia*, *Fibrobacter*, *Treponema*, *Sphaerocheata*, *Phasolarctobacterium*, *Christensenellaceae R-7 group*, *Lachnoclostridium*, *Cellulosilyticum, Marvinbryantia, Coprostanoligenes group, Lachnospiraceae XPB1014, Lachnospiraceae UCG-009, Erysipelatoclostridium*, *Clostridium sensu stricto 1*, and *Bacteroidales BS11 gut group* as well as *Family XIII AD3011 group* of *Anaerovoracaceae*, the *UCG-004* genus within *Erysipelatoclostridiaceae*, the *UCG-002* and *UCG-005* genuses of *Oscillospiraceae*, and the *UCG-010* genus and family of *Oscillospirales*.

Fecal pH was positively correlated with five BCG (in addition to BCG_300) (r_s_ ≥ |0.30|; *p* ≤ 0.05), which contained ASV from taxa including *Anaerovoracaceae*, the *Family XIII AD3011 group* of *Anaerovoracaceae*, *Akkermansia*, *Christensenellaceae R-7 group*, *Fibrobacter*, *Treponema*, *Catenisphaera*, *Alloprevotella*, *Bacteroidales RF16 group*, *Marvinbryantia*, *Coprostanoligenes group*, *Bacteroidales UCG-001*, the *NK4A214 group* of *Oscillospiraceae*, and the *WCHB1-41* genus, family, and order within *Kiritimatiellae*. Fecal pH was negatively correlated with BCG_94. This BCG included ASV assigned to *Prevotella*, *Sarcina*, and *Clostridium sensu stricto 1*.

### 3.6. Relationships between Fecal Microbiota and Forage Nutrients

Application of random forest regressors demonstrated that forage nutrient concentrations could be predicted based on bacterial community composition (BCG composition of the full microbial community), including water-soluble carbohydrate (WSC) and NSC at a predictive accuracy > R^2^ = 0.50 and crude protein (CP) with a predictive accuracy of R^2^ = 0.41 (*p* < 0.0001; Table 7). Weaker, but still statistically significant, predictive accuracy was found for digestible energy (DE), acid detergent fiber (ADF), neutral detergent fiber (NDF), ethanol-soluble (ESC), and starch (*p* < 0.04).

Similar to results for fecal metabolites, when random forest regressors were applied only to the top 25 BCG identified as most predictive of forage type, model accuracies improved (Table 7). Forage CP, NSC, and WSC remained as the nutrients most accurately predicted by the bacterial composition of this subset of BCG, all with R^2^ > 0.60 (*p* < 0.0001). Model predictive capacity with the reduced feature set was also above R^2^ = 0.40 for starch; weaker, but statistically significant, predictive accuracy was found all other nutrients (*p* < 0.0001). 

**Figure 5:**
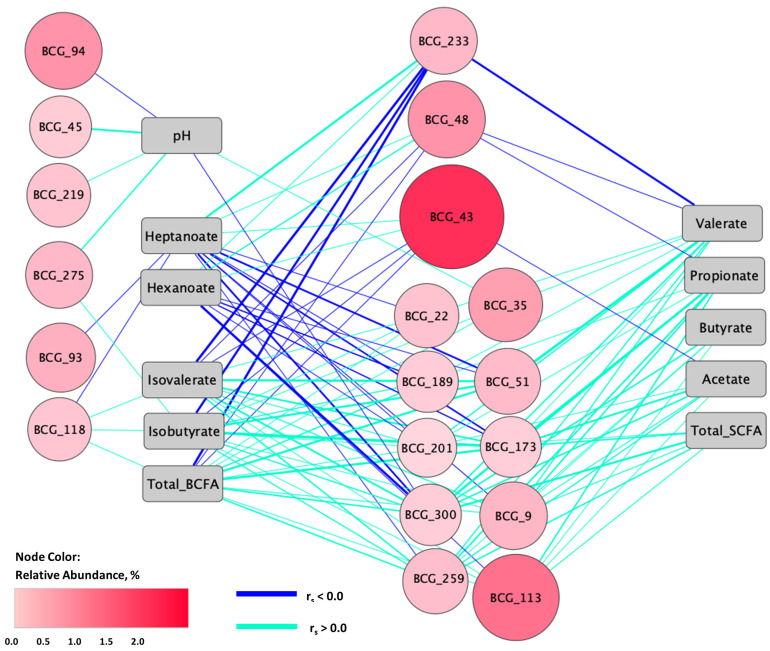
Network map of bacterial co-abundance groups (BCG) and fermentation metabolites as well as fecal pH. Correlations were evaluated between BCA most predictive of forage type (based on random forest modeling of BCG composition) and fecal pH, short-chain fatty acids (SCFA) and branched-chain fatty acids (BCFA). In addition to node color, node size was also mapped to BCG relative abundance. Line width is mapped to correlation strength, with wider lines indicating stronger correlations between nutrients and BCG. Significant Spearman correlations (r_s_ ≥ |0.30|; *p* ≤ 0.05) were included in the network map.

Subsequent correlation analysis found 23 BCG (of the 25 in the reduced feature set) were correlated with at least one forage nutrient (Spearman correlation; r_s_ ≥ |0.30|; *p* ≤ 0.05; Figure 5). Nine BCG were correlated with ADF, NDF, CP, and DE. In all cases, opposite correlations were seen between ADF/NDF and CP/DE (i.e., if ADF and NDF were positively correlated with a BCG, CP and DE were negatively correlated). There were distinct ASV from the taxa *Christensenellaceae R-7 group* and *NK4A214 group* of *Oscillospiraceae* that were members of separate BCG which were positively and negatively responding to these nutrients (i.e., these taxonomic classifications were found across all positive and negative responder groups). The BCG negatively correlated with ADF/NDF, and thus positively correlated with CP/DE included ASV members assigned to *Clostridium butyricum* and genera *Papillibacter*, *Lachnoclostridium*, *Cellulosilyticum*, *Fibrobacter*, *Treponema*, *Catenisphaera*, *Bacteroidales RF16 group*, *Synergistaceae*, *Mogibacterium*, *Rikenellaceae RC9 gut group*, the *Hallii group* of *Lachnospiraceae*, the *p-251-o5* genus and family of *Oscillospiraceae*, and the *F082* genus and family of *Bacteroidales*, as well as multiple ASV assigned only to the family level for *Lachnospiraceae*. The BCG positively correlated with ADF/NDF and negatively correlated with CP/DE included ASV within *Sphaerochaeta*, *Phascolarctobacterium*, *Bacteroidales UCG-001*, *Rikenellaceae RC9 gut group*, *Coprostanoligenes group*, the *UCG-002* genus within *Oscillospiraceae*, and the *UCG-010* genus and family within *Oscillospirales*. Four additional BCG were correlated with some combination of ADF/NDF and CP/DE. The BCG negatively correlated with ADF/NDF, and thus positively correlated with CP/ DE contained ASV members assigned to additional taxa including *Ruminococcus*, *Anaerovibrio*, *Pseudobutyvibrio*, *Lachnospiraceae UCG-009*, *probable genus 10* of *Lachnospiraceae*, and the *WCHB1-41* genus, family and order of *Kiritimatiellae*. The BCG positively correlated with ADF/NDF, and negatively correlated with CP/DE included ASV within *Bacteroidales BS11 gut group* and *Clostridium sensu stricto 1*. Forage ADF and NDF were also negatively correlated with BCG_300 (*Streptococcus* and *Lachnospiraceae XPB1014*), but this BCG was not correlated with either CP or DE. Forage CP was also positively correlated with BCG_35, for which there was no relationship with ADF, NDF, or DE, but for which there was also a negative correlation with NSC and WSC. The ASV members of BCG_35 were assigned to taxa including *Akkermansia*, *Fibrobacter*, *Treponema*, *Christensenellaceae R-7 group*, and *Family XIII AD3011* within *Anaerovoracaceae*.

Forage NSC and WSC were correlated with 13 and 12 BCG, respectively (r_s_ ≥ |0.30|; *p* ≤ 0.05). In addition to the above-mentioned taxa from ASV within BCG_35, ASV within BCG negatively correlated with both NSC and WSC were assigned to *Lactobacillus equigenerosi* and genera including *Lachnoclostridium*, *Cellulosilyticum*, *Catenisphaera*, *Marvinbryantia*, *Erysipelatoclostridium*, *Mogibacterium*, *Lachnospiraceae XPB1014 group, Lachnospiraceae UCG-009*, *Coprostanoligenes group*, *UCG-005 metagenome* within *Oscillospiraceae*, the *Hallii group* within *Lachnospiraceae*, the *NK4A214 group* of *Oscillospiraceae*, the *Incertae Sedis* genus of *Ethanoligenenaceae*, the *WCHB1-41* genus, family, and order within *Kiritimatiellae*, as well as *Denitrobacterium detoxificans*. Additional ASV were assigned only to the family level for *Synergistaceae*, *Lachnospiraceae*, *Oscillospiraceae*, and *Anaerovoracaceae*. Forage NSC and WSC were positively correlated with BCG_94 (*Clostridium sensu stricto 1*, *Prevotella,* and *Sarcina*). Forage ESC was correlated with 9 of the same BCG as NSC and WSC, but also had a negative correlation with BCG_173, which contained multiple ASV assigned to *Lachnospiraceae*, *probable genus 10* within *Lachnospiraceae*, *Pseudobutyvibrio* as well as to the *p-251* genus and family of *Bacteroidales*. Starch was positively correlated with BCG_113 and BCG_300 and negatively correlated with two BCG, BCG_43 and BCG_48, containing ASV from *Sphaerochaeta*, *Phascolarctobacterium*, *Christensenellaceae R-7 group*, *Coprostanoligenes group*, *Bacteroidales UCG-001*, *Rikenellaceae RC9 gut group*, the *NK4A214 group* of *Oscillospiraceae*, the *UCG-002* genus within *Oscillospiraceae*, and the *UCG-010* genus and family within *Oscillospirales*; these BCG were also among those positively correlated with ADF/NDF and negatively correlated with CP/DE. 

**Figure 6:**
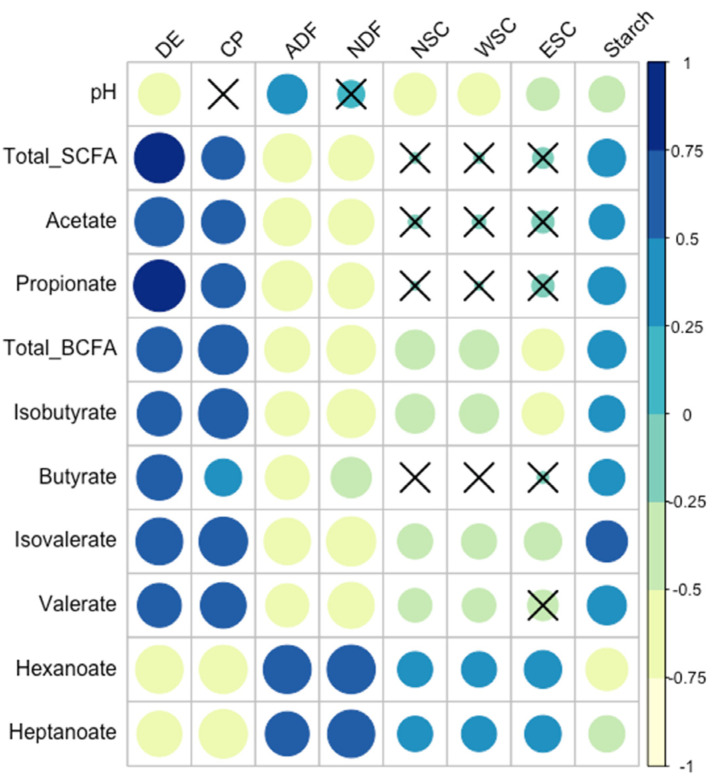
Correlation of forage nutrients and fermentation metabolites. Nutrients included digestible energy (DE), crude protein (CP) acid detergent fiber (ADF), neutral detergent fiber (NDF), non-structural carbohydrate (NSC), water-soluble carbohydrate (WSC) and ethanol-soluble carbohydrate (ESC). Significance of Spearman correlations was set at r_s_ ≥ |0.30| (*p* ≤ 0.05). Cells with an “X” indicate non-significant correlations. Color and size of circles within cells are mapped to direction and strength of correlation. Larger, darker blue circles indicate a stronger positive correlation (closer to r_s_ = 1.0); larger, lighter yellow circles indicate a stronger negative correlation (closer to r_s_ = −1.0).

Relationships between fecal variables and forage nutrients were also evaluated through correlation analysis (Figure 6). Acetate, butyrate, propionate, valerate, isobutyrate, isovalerate and total SCFA and BCFA were positively correlated with DE (Spearman correlation; r_s_ ≥ 0.62) and CP (r_s_ ≥ 0.41), but were negatively correlated with NDF (rs ≤ −0.48) and ADF (r_s_ ≤ −0.57; *p* ≤ 0.008). There was also a weaker positive correlation between these fecal metabolites and starch (r_s_ ≥ 0.39; *p* ≤ 0.01). Total BCFA, isobutyrate, and isovalerate were negatively correlated with NSC, WSC, and ESC (r_s_ ≤ −0.37; *p* ≤ 0.02), with a weaker negative correlation between valerate and NSC and WSC (r_s_ ≤ −0.34; *p* ≤ 0.03). Hexanoate and heptanoate were negatively correlated with DE, CP, and starch (r_s_ ≤ −0.39; *p* ≤ 0.01), but were positively correlated with NDF, ADF, NSC, WSC, and ESC (r_s_ ≥ 0.37; *p* ≤ 0.02). Conversely, fecal pH was positively correlated with ADF (r_s_ = 0.47; *p* = 0.002), but negatively correlated with DE, NSC, WSC, ESC, and starch (r_s_ ≤ −0.32; *p* ≤ 0.04).

### 3.7. Blood Samples and Glycemic/Insulinemic Responses

**Figure 7:**
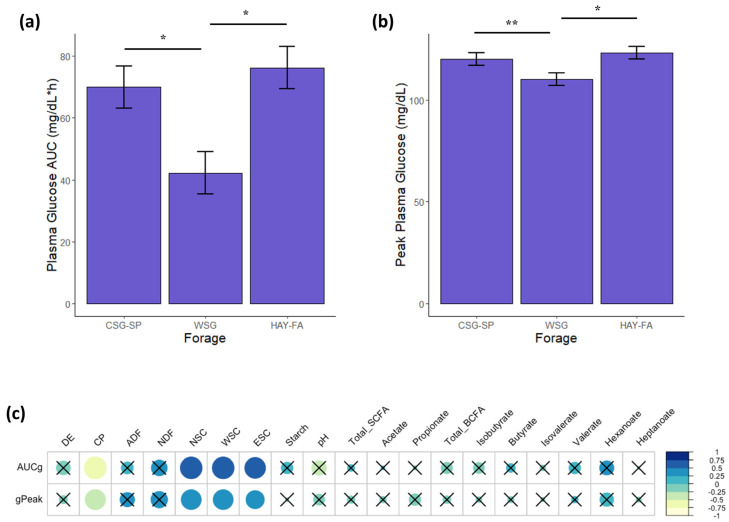
Plasma glucose responses to oral sugar tests and relationship with forage nutrients and fermentation metabolites. Oral sugar tests were administered to horses adapted to spring cool-season grass pasture (CSG-SP) and warm-season grass in the summer slump period (WSG) in addition to a standardized hay diet in the fall at the end of the grazing season (HAY-FA). Glycemic responses including the positive incremental area under the curve (**a**) and peak plasma glucose (**b**) are presented as least squares means ± SEM. A single asterisk indicates differences between forages at *p* ≤ 0.05 (mixed model ANOVA with Tukey’s post hoc adjustment). A double asterisk indicates a trend for differences at *p* ≤ 0.10. (**c**) Correlated nutrients included digestible energy (DE), crude protein (CP) acid detergent fiber (ADF), neutral detergent fiber (NDF), non-structural carbohydrate (NSC), water-soluble carbohydrate (WSC) and ethanol-soluble carbohydrate (ESC). Fecal metabolites included short- and branched-chain fatty acids. Significance of Spearman correlations was set at r_s_ ≥ |0.30| (*p* ≤ 0.05). An “X” indicates non-significant correlations. Color and size of circles within cells are mapped to direction and strength of correlation. Larger, darker blue circles indicate a stronger positive correlation (closer to r_s_ = 1.0); larger, lighter yellow circles indicate a stronger negative correlation (closer to r_s_ = −1.0).

Plasma glucose responses to OST administration differed by forage (mixed model ANOVA with Tukey’s post hoc adjustment; *p* ≤ 0.01). The AUC for glucose was lowest when horses were adapted to WSG (42.4 ± 6.8 mg/dL*h) vs. CSG-SP (70.0 ± 6.8 mg/dL*h) and HAY-FA (76.3 ± 6.8 mg/dL*h; *p* ≤ 0.03; Figure 7a). Peak plasma glucose was also lower for WSG (110 ± 3 mg/dL) in comparison to HAY-FA (123 ± 3 mg/dL; *p* = 0.01), and there was a trend for lower peak plasma glucose for WSG than for CSG-SP (120 ± 3 mg/dL; *p* = 0.06; Figure 7b). 

Forage did not, however, impact OST insulin responses. Neither AUC (CSG-SP: 36.1; WSG: 29.1; HAY-FA: 32.7 ± 6.4 mIU/L*h) or peak plasma insulin (CSG-SP: 28.8; WSG: 25.5; HAY-FA: 26.6 ± 3.3 mIU/L) varied by forage (Figure S2). Fasting plasma insulin (CSG-SP: 3.69; WSG: 5.04; HAY-FA: 5.20 ± 0.64 mIU/L) and glucose (CSG-SP: 81.4; WSG: 78.7; HAY-FA: 81.3 ± 1.4 mg/dL) also did not differ by forage (Figure S3). 

There were trends for differences by forage in proxies for insulin sensitivity including the fasting glucose-to-insulin ratio (FGIR) and reciprocal of the square root of insulin (RISQI; mixed model ANOVA with Tukey’s post hoc adjustment; *p* ≤ 0.10; Figure S4). Differences in FGIR and RISQI were limited to WSG (FGIR: 25.2 ± 16.4; RISQI: 0.58 ± 0.04) vs. HAY (FGIR: 16.4 ± 2.1; RISQI: 0.46 ± 0.04; *p* = 0.08). However, the modified insulin-to-glucose ratio (MIRG), a proxy for the insulin secretory response, did not vary by forage (Figure S4).

### 3.8. Relationships between Glucose Metabolism and the Fecal Microbiota

Glucose and insulin dynamics were only assessed after three of the forages (CSG-SP, WSG, and HAY-FA), and this smaller dataset precluded the use of random forest regression modelling for these variables. As the primary metabolic differences in grazing horse metabolism were AUC and peak plasma glucose in response to OST administration, correlation analysis was conducted to explore relationships between these metabolic variables and BCG. Only one BCG (of the 25 BCG in the reduced feature set) was correlated with AUC (r_s_ = −0.48; *p* = 0.04), with members of BCG_124 including ASV within *Papillibacter*, *Christensenellaceae R-7 group*, and *Synergistaceae*. Four additional BCG were correlated with peak plasma glucose. Positive correlations were found between peak glucose and BCG_51 and BCG_305 (r_s_ ≥ 0.43; *p* ≤ 0.04). These BCG included ASV mapped to genera including *Lachnospiraceae XPB1014*, *Lachnospiraceae UCG-009*, *Erysipelatoclostridium*, *Catenisphaera*, and *Fibrobacter* as well to the family level for *Anaerovoracaceae*. Conversely, BCG_113 (*Clostridium butyricum*, *Papillibacter*, *Bacteroidales RF16 group*) and BCG_259 were negatively correlated with peak glucose (r_s_ = −0.44; *p* = 0.03). Members of BCG_259 included ASV assigned at the family level to *Lachnospiraceae* in addition to the *Hallii group* within *Lachnospiraceae*. 

While relationships between AUC and peak plasma glucose and forage nutrients were identified through correlation analysis (Spearman correlation; r_s_ ≥ |0.41|; *p* ≤ 0.04; Figure 7c), these metabolic variables were not correlated with any fecal variables including SCFA, BCFA, and pH. Forage NSC, WSC, and ESC were positively correlated with AUC (r_s_ ≥ 0.53; *p* ≤ 0.007) and peak glucose (r_s_ ≥ 0.41; *p* ≤ 0.04). There was also a negative correlation between CP and glucose responses to OST administration (r_s_ ≤ −0.50; *p* ≤ 0.01). Forage DE, NDF, ADF, and starch, however, were not correlated with AUC or peak glucose.

## 4. Discussion

### 4.1. Forage Type and the Microbiome

Warm-season grasses can be utilized to bridge the “summer slump” forage gap in cool-season grass grazing systems [1,2], and differences in NSC between these forage types could have implications for equine metabolic health [1,5,9]. However, there is limited information on the impacts of grazing warm-season grasses on the equine hindgut microbiome as well as potential associations between forage nutrients, the hindgut microbiome, and equine metabolic responses. Therefore, this study aimed of to characterize the fecal microbiota of horses adapted to different forage types and to explore relationships between forage nutrients, microbial composition, fecal metabolites, and glycemic responses of grazing horses. Results of this study clearly demonstrated that shifts in fecal microbiome structure and species composition occur as horses are adapted to different forages within an integrated warm- and cool-season grass rotational grazing system. This was supported by statistical differences in both α- and β-diversity. Furthermore, random forest classification modelling was able to predict forage type based on microbial community composition, indicating the influence of forage type on the fecal microbiome. Finally, forage-specific BCG were identified through LEfSe analysis, but the 25 BCG identified as most predictive of forage type through random forest modeling and subsequently analyzed by LEfSe represented only ~10% of the total fecal microbiota across all forages. This indicates that distinct and identifiable shifts in microbial composition do occur as horses adapt to different forage types. However, the majority of the microbiome (~90% in the current study) is resistant and/or resilient to potential perturbations induced by transitioning among forage types with different physical and chemical properties, including between cool-season and warm-season grass pastures.

The BCG identified as markers specific to HAY-SP included multiple ASV assigned to taxa to which fibrolytic and butyrate-producing functions have been previously ascribed [74,75,76]. The BCG specific to this forage contained a heavy representation of ASV within the *Lachnospiraceae* family as well as *Fibrobacter*, *Ruminococcus*, and *Pseudobutyvibrio*. Prior studies have also reported increased prevalence of *Lachnospiraceae* in horses fed hay vs. pasture [19,77]. Conversely, ASV assigned to *Anaerovibrio* were also identified in BCG markers of HAY-SP. Increases in this genus in response to abrupt inclusion of dietary starch have been documented [78] as well as in temporal proximity to oral administration of oligofructose in experimental laminitis induction models [79,80]. The co-occurrence of bacteria in these taxonomic groups could potentially be reflective of the nutrient composition of HAY-SP, which was the highest in both fiber and NSC of all forages. Co-occurrence of these bacteria may also reflect cross-feeding relationships between bacteria and/or metabolic plasticity of bacterial populations. These factors may also account for unexpected associations revealed by analysis in the current study, such as ASV assigned to *Streptococcus* within BCG markers of HAY-FA, despite the relatively low NSC content of this forage. However, Zhu et al. [77] also reported a lower abundance of species within *Streptococcaceae* in horses maintained on pasture vs. horses fed hay or silage [77].

Overall, the fecal microbiota of horses adapted to cool-season pasture was characterized by bacteria capable of utilizing rapidly fermentable fibers, which could explain the greater fecal SCFA concentrations for CSG-SP and CSG-FA. Capacity for hemicellulose fermentation and SCFA production have been previously documented in the *Bacteroidales BS11 gut group* [81], which was identified only in BCG markers of CSG-SP. Equine studies have found increased relative abundance of *Bacteroidales BS11 gut group* in horses fed barley vs. a hay diet [82] and in horses presenting with colic [83,84]. For CSG-FA, BCG markers included genera such as *Alloprevotella* and *Erysipelatoclostridium*, which are also associated with degradation of fermentable fibers [85,86,87]. The BCG markers of CSG-FA also included ASV assigned *WCHB1-41* clade within *Kiritimatiellae*. Prior studies have found enrichment of the phylum *Kiritimatiellaeota* in the fecal microbiota of horses with equine metabolic syndrome and insulin dysregulation [26,27]. In contrast to results of the present study, Fitzgerald et al. [27] reported increased abundance of this phylum in response to a change from pasture to a hay diet in both healthy and insulin dysregulated ponies, and Ericsson et al. [88] similarly found increased abundance of the class *Kiritimatiellae* when horses with equine metabolic syndrome were transitioned from pasture to a hay diet. These conflicting results reinforce that nutritional composition of specific forages, rather than form of forage alone, need to be considered when evaluating interstudy effects of diet on the equine microbiome.

A number of interesting relationships were found between BCG identified as markers specific to WSG and fecal variables, forage nutrients, and horse glucose metabolism. Warm-season grasses are characteristically lower in soluble carbohydrates than cool-season grasses [1,5], and of all forages evaluated in the current study, NSC and WSC were lowest in WSG. Four of the five BCG markers of WSG were negatively correlated with forage NSC and WSC, including BCG_35, which contained ASV assigned *Akkermansia* as well as *Fibrobacter*, *Treponema*, *Christensenellaceae R-7 group*, and the *Family XIII AD3011 group* of *Anaerovoracaceae. Akkermansia* has been linked to metabolic health via regulation of inflammatory responses and has also been explored for probiotic use in animal species [89,90,91,92]. Lindenberg et al. [93] recently demonstrated immune (and inflammatory) modulation by specific hindgut microbiota in horses, including *Akkermansia* spp. Furthermore, supplementation with mannanoligosaccharides and fructooligosaccharides led to increased abundance of *Akkermansia muciniphilia* in a subsequent study [94], and improvements in insulin sensitivity have been previously documented in horses supplemented with low doses of fructooligosaccharide [95,96]. 

Markers of WSG also included BCG_113, which contained an ASV assigned to *Clostridium butyricum*. *Clostridium butyricum* is a prolific butyrate producer that has also been investigated for probiotic use due to its capacity to promote anti-inflammatory responses and improve gut barrier function and metabolic health [97,98,99]. BCG_35 was positively correlated with CP as well as isobutyrate and total BCFA, which reflects the proteolytic capacity of this BCG. BCG_113 was also positively correlated with total SCFA and all three major SCFA (acetate, butyrate, and propionate) in addition to isobutyrate, isovalerate, and total BCFA. This BCG was also negatively correlated with peak plasma glucose. These findings suggest that this bacterial group including *Clostridium butyricum* could play a role in modulation of equine metabolic health. While limited connections between the gut microbiota and metabolic responses were observed in the current study, three of the five BCG correlated with AUC and peak plasma glucose were WSG-specific BCG markers. Further research is necessary to determine if other WSG species or varieties would produce similar effects as those observed in the current study.

While *Akkermansia* and *Clostridium butyricum* have been investigated in mice and other species, comparatively little research has been conducted to understand the function of these bacteria in the equine hindgut. The relationships found between ASV in these taxa and forage nutrients, fecal metabolites, and equine glycemic responses in addition to associations with low-NSC warm-season grasses in the current study support further research to determine the role of these bacteria, factors including dietary interventions that can promote prevalence in the hindgut, and potential probiotic applications.

### 4.2. Forage Nutrients and the Microbiome

Results of this study also confirmed the strong influence of dietary nutrients on the equine microbiome and provided insights into the complex relationships between forage nutrients and the gut microbiota. The concentrations of several nutrients could be predicted based on microbial community composition. Furthermore, regression model accuracy improved when the reduced feature set identified through prior forage classification modeling was utilized for prediction of nutrient concentrations. This indicates that differences in forage nutrients were driving the shifts in equine fecal microbial communities as horses adapted to various forages within the integrated grazing system. Results of this study revealed the influence of soluble carbohydrates including NSC and WSC on fecal microbial community composition. In contrast, fiber was not identified as a key nutrient shaping differences in microbial communities. Forage NSC and WSC concentrations could be predicted based on microbial community composition with an accuracy of R^2^ = 0.61 and R^2^ = 0.67, respectively, while predictive accuracy for ADF and NDF was < 0.40 (R^2^). Random forest modeling also revealed that in addition to soluble carbohydrates, the gut microbiota were also influenced by CP (R^2^ = 0.62). The impact of CP on the gut microbial community was interesting, but unsurprising, as two-thirds to three-fourths of total tract nitrogen digestion and absorption occurs in the equine hindgut [100], and many microbial species are capable of proteolytic fermentation [101]. 

Prior studies in horses have found distinct effects of high-fiber vs. lower-fiber diets on the hindgut microbial community, with benefits of increased fiber including greater microbial diversity [102], reduction of lactic-acid producing bacteria [101,103], and less-acidic hindgut pH [104,105]. However, these previous studies have been primarily conducted in horses fed concentrate vs. forage-based diets in which there was broad variation in fiber concentrations between treatments. In the current study, horses were maintained on a forage-only diet throughout, and there were relatively high concentrations of NDF and ADF found across all forages. It is possible that only minimal shifts occur in bacterial populations most responsive to fiber above a certain concentration threshold, and that these populations remained relatively stable throughout the study (and thus fiber concentration was not able to be predicted with strong accuracy by random forest modeling). However, it should be noted that the concentrations of NSC, WSC, and CP in forages is low in comparison to that of fiber. Additionally, in contrast to fiber, these nutrients are all subject to digestion in the equine foregut, and only a fraction of the total soluble carbohydrates and CP ingested would be available for bacterial fermentation in the hindgut. Thus, only a small amount of these nutrients were capable of exerting influence on gut microbial communities. Digestibility was not evaluated in the current study, however, and a more in-depth analysis of total digestibility and digestibility of specific nutrients would be necessary to fully understand the interactions between forage nutrients and the gut microbiota.

### 4.3. Fecal pH and Fermentation Metabolites

Fecal pH is commonly utilized as a marker of microbial activity in the equine hindgut [20,103]. Differences in fecal pH in the current study indicated that functional changes occur in the equine gut microbiota, in addition to shifts in microbiome structure and composition, as horses adapt to different forages. Fecal pH was highest in horses adapted to WSG and HAY-FA, which were lower in NSC than CSG-SP, CSG-FA. Accordingly, fecal pH was negatively correlated with forage NSC. Lower fecal pH is broadly associated with hindgut dysfunction in horses [106,107], and thus the higher fecal pH in horses adapted to WSG in comparison to cool-season pasture could suggest some benefit to grazing horses on warm-season grass pastures. However, mean pH was above 6.5 for CSG-SP, CSG-FA, and HAY-SP, which is within ranges previously documented in healthy forage-fed horses [33,108]. Therefore, while statistically significant, the difference in fecal pH between horses adapted to WSG vs. cool-season pasture may not be of physiological relevance.

Differences in fermentation metabolites across forages did not mirror results for fecal pH, as differences between forages for SCFA and BCFA were primarily between pasture forages and the hay diets. Numerically, SCFA and BCFA concentrations in horses adapted to WSG were intermediate between the cool-season pasture (greatest) and hay (lowest), but these differences were not statistically significant. Furthermore, while statistically significant, the predictive accuracy of random forest regressors for fecal metabolites and pH were lower in comparison to those found for forage nutrients. Numerous studies have noted that while the gastrointestinal tract harbors a phylogenetically diverse community, many unrelated microorganisms are capable of performing similar functions [109,110,111,112,113]. The relatively poor predictive accuracy of regressors for fecal metabolites in the current study is likely due to this functional redundancy within the equine microbial community. Functional redundancy has been previously suggested as a contributing factor in stability of the gut microbial communities [111,113] including within the equine microbiome during transitions between warm- and cool-season grasses [24]. 

### 4.4. Glucose and Insulin Metabolism

While there were distinct shifts in the fecal microbiota across forages in the integrated system, forage type exerted minimal effects on glucose metabolism. A large body of research, primarily conducted in mouse models, has established that diet (and dietary intervention) modulates metabolic health in a microbiome-dependent manner [29,30,114]. However, shifts in gut microbial structure and function may precede changes in metabolic outcomes [115]. The adaptation period utilized in the present study was of a similar duration as used in prior studies evaluating the microbiome of forage-fed horses [22,23,116], and is considered sufficient for stabilization of microbial communities [95]. However, longer treatment periods are often required to assess metabolic adaptations to diet [13,103,117,118]. The lower AUC and peak plasma glucose in horses adapted to WSG in the current study does suggest that glucose may be cleared more rapidly in horses adapted to this forage, but a longer adaptation period would be necessary to confirm these results. Regardless, glucose and insulin responses to the OST for all forages evaluated in the current study were within normal and previously reported ranges [12,34], indicating that substantial metabolic changes are unlikely within the context of integrated rotational grazing management even with the observed changes in the fecal microbiota, fecal metabolites, and pH. Additionally, the lack of correlation between any fermentation metabolites and glucose responses, as well as the relatively small number of BCG correlated with AUC and peak plasma glucose, suggest that any difference in glycemic responses of horses in the current study may not have been heavily dependent on shifts in the hindgut microbiome.

### 4.5. Additional Considerations

Seasonal and environmental factors should be considered when interpreting results of this study. Prior studies have reported seasonal changes in microbial diversity and species composition [119,120,121] but also lacked a true seasonal control, and thus, the effect of seasonality on the equine hindgut microbiome requires further investigation. Seasonal variance in insulin sensitivity has been documented in grazing horses [15,122], but when fed controlled diets without nutrient fluctuations seen in pasture forages, minimal seasonal differences in circulating glucose and insulin and insulin sensitivity have been found in healthy horses [15,123,124]. Environment and management factors beyond season can also impact pasture forage nutrient composition. Characterizing the microbiome of a larger number of grazing horses over multiple years and in multiple locations/regions would allow a more robust evaluation of the impacts of cool- versus warm-season grasses on the equine hindgut microbiome.

Finally, it should be noted that the analytical approach in this study differs from more conventional taxon-based analysis. Rather, this study implemented a guild-based approach, grouping individual microbial ASV by co-abundance to subsequent analysis of abundance profiles [123,124,125,126,127]. This strategy has been previously utilized as an alternative to grouping bacteria by taxonomy [24,29,30,92,121]. Substantial genetic variation is possible within taxa, even at the species level, and therefore bacteria with similar taxonomic assignments may not represent a functionally homologous group [29,92,127]. Results of the current study also illustrate this concept, as ASV with the same assigned taxonomy were found in BCG characteristic of different forages as well as in separate BCG that positively and negatively responded to forage nutrients and with both positive and negative relationships with fecal metabolites and grazing horse metabolism.

## 5. Conclusions

In conclusion, distinct shifts in equine fecal microbial community structure and composition occur as horses adapt to different forages within an integrated warm- and cool-season grass rotational pasture system, but a substantial impact of this management practice on glucose metabolism in healthy adult grazing horses is unlikely. Forage NSC, WSC, and CP were the most influential nutrients driving these shifts in microbial composition. Results of this study underscore the potential for relatively small amounts of NSC to influence hindgut microbial composition and also that protein utilization may be an important ecological niche within the microbiome of forage-fed horses. Fecal BCFA and SCFA concentrations were higher in horses adapted to all pasture forages versus hay, but in comparison to forage nutrients, bacterial community composition did not have as strong an impact on fermentation metabolites, likely reflecting functional redundancy of the microbial community. The guild-based analytical approach utilized in this study also identified key relationships between specific bacterial groups associated with adaptation to warm-season grass pasture and forage nutrients, fecal metabolites, and equine glycemic responses to administration of oral sugar tests. Relationships identified in this study revealed new insights and targets for future research necessary to better understand the function of *Akkermansia* spp. and *Clostridium butyricum* in the hindgut microbiome of grazing horses, as these bacteria may play a role in modulation of equine metabolic health.

## Figures and Tables

**Table 1:** Nutrient composition ^1^ of hay diets and pasture forages.

Nutrients ^2,3^	Forage ^4^							
	HAY-SP	CSG-SP		WSG		CSG-FA		HAY-FA
		BRS	CRS	BRS	CRS	BRS	CRS	
**DE, Mcal/kg**	2.02	2.18	2.06	2.15	2.03	2.34	2.22	2.00
**CP, %**	5.6	13.1	16.5	21.4	22.7	23.7	21.5	10.2
**ADF, %**	40.9	35.3	36.4	31.4	36.9	30.5	30.0	42.2
**NDF, %**	66.1	59.4	62.1	58.7	61.8	50.5	55.4	65.8
**NSC, %**	18.4	12.4	8.5	7.1	2.6	11.3	11.5	9.1
**WSC, %**	18.0	10.3	7.4	4.8	2.3	10.2	10.2	8.1
**ESC, %**	7.0	6.6	3.7	4.0	1.9	8.3	6.9	6.9
**Starch, %**	0.40	2.07	1.17	2.30	1.37	1.13	1.23	1.00

^1^ Determined by near-infrared spectroscopy (Equi-Analytical Laboratories, Ithaca, NY, USA). ^2^ Nutrient concentrations are reported on a dry-matter basis as means of three representative samples collected per forage. ^3^ Abbreviations: digestible energy (DE), crude protein (CP), neutral detergent fiber (NDF), acid detergent fiber (ADF), non-structural carbohydrates (NSC), water-soluble carbohydrates (WSC), ethanol-soluble carbohydrates (ESC). ^4^ HAY-SP: initial standardized cool-season grass hay diet in the spring; CSG-SP: cool-season grass pasture in the spring; WSG: warm-season grass, either crabgrass or bermudagrass, in the summer slump period; CSG-FA: cool-season pasture in the fall; HAY-FA: final standardized cool-season grass hay diet in the fall.

**Table 2:** 16S rRNA gene sequence read counts before and after filtering ^1^.

Step	Statistic	Count
**Initial**	Total	881,189
**Quality and Chimera Filtering ^2^**	Total	730,065
	Minimum sample^−1^	12,460
	Maximum sample^−1^	30,170
	Mean sample^−1^	18,252
	Median sample^−1^	18,890
**Removal of Low Abundance Features**	Total	648,797
	Minimum sample^−1^	10,989
	Maximum sample^−1^	26,948
	Mean sample^−1^	16,220
	Median sample^−1^	15,587

^1^ Filtering was conducted in Qiime 2 (v.2020.8) [40]. ^2^ Filtering was conducted using DADA2 [43,44].

**Table 3:** Bacterial co-abundance groups (BCG) ^1^ identified as markers ^2^ of standardized hay diets.

Forage ^2^	BCG ^3^	LDA ^4^	ASV Taxonomic Lineage ^5^
**HAY-SP**	BCG_9	4.55	*Firmicutes | Clostridia | Oscillospirales | Oscillospiraceae | Papillibacter | uncultured_Clostridiales* *Bacteroidota | Bacteroidia | Bacteroidales | Rikenellaceae | Rikenellaceae_RC9_gut_group | uncultured_bacterium* *Firmicutes | Clostridia | Christensenellales | Christensenellaceae | Christensenellaceae_R-7_group | uncultured_Clostridia* *Firmicutes | Clostridia | Lachnospirales | Lachnospiraceae (2 ASV)* *Firmicutes | Clostridia | Oscillospirales | Oscillospiraceae | NK4A214_group | uncultured_rumen*
	BCG_18	5.03	*Firmicutes | Clostridia | Oscillospirales | Ruminococcaceae | Ruminococcus | Ruminococcus_*sp. *Firmicutes | Clostridia | Lachnospirales | Lachnospiraceae (3 ASV)* *Firmicutes | Clostridia | Lachnospirales | Lachnospiraceae | Lachnospiraceae_UCG-009 | uncultured_bacterium* *Firmicutes | Clostridia | Christensenellales | Christensenellaceae | Christensenellaceae_R-7_group | uncultured_prokaryote*
	BCG_93	4.55	*Bacteroidota | Bacteroidia | Bacteroidales | p-251-o5 | p-251-o5 | uncultured_bacterium* *Firmicutes | Clostridia | Lachnospirales | Lachnospiraceae* *Firmicutes | Clostridia | Lachnospirales | Lachnospiraceae | probable_genus_10 | uncultured_bacterium* *Firmicutes | Clostridia | Lachnospirales | Lachnospiraceae | Pseudobutyrivibrio*
	BCG_116	4.52	*Bacteroidota | Bacteroidia | Bacteroidales | p-251-o5 | p-251-o5 | uncultured_bacterium* *Firmicutes | Negativicutes | Veillonellales-Selenomonadales | Selenomonadaceae | Anaerovibrio | uncultured_bacterium* *Firmicutes | Clostridia | Oscillospirales | Ruminococcaceae | Ruminococcus*
	BCG_201	4.28	*Firmicutes | Clostridia | Oscillospirales | Oscillospiraceae | NK4A214_group* *Bacteroidota | Bacteroidia | Bacteroidales | p-251-o5 | p-251-o5 | uncultured_bacterium*
	BCG_305	4.27	*Firmicutes | Bacilli | Erysipelotrichales | Erysipelotrichaceae | Catenisphaera | uncultured_bacterium* *Fibrobacterota | Fibrobacteria | Fibrobacterales | Fibrobacteraceae | Fibrobacter | uncultured_bacterium*
**HAY-FA**	BCG_22	4.67	*Firmicutes | Clostridia | Lachnospirales | Lachnospiraceae* *Firmicutes | Bacilli | Lactobacillales | Lactobacillaceae | Lactobacillus | Lactobacillus_equigenerosi* *Firmicutes | Clostridia | Oscillospirales | [Eubacterium]_coprostanoligenes_group | [Eubacterium]_coprostanoligenes_group* *Firmicutes | Clostridia | Oscillospirales | Oscillospiraceae | uncultured*
	BCG_118	4.56	*Firmicutes | Bacilli | Erysipelotrichales | Erysipelatoclostridiaceae | UCG-004 | uncultured_rumen* *Firmicutes | Clostridia | Lachnospirales | Lachnospiraceae | Marvinbryantia* *Firmicutes | Clostridia | Oscillospirales | Oscillospiraceae | UCG-005 | uncultured_bacterium*
	BCG_173	4.20	*Spirochaetota | Spirochaetia | Spirochaetales | Spirochaetaceae | Treponema | uncultured_bacterium* *Bacteroidota | Bacteroidia | Bacteroidales | F082 | F082 | uncultured_bacterium* *Bacteroidota | Bacteroidia | Bacteroidales | Rikenellaceae | Rikenellaceae_RC9_gut_group | uncultured_bacterium*
	BCG_300	4.87	*Firmicutes | Clostridia | Lachnospirales | Lachnospiraceae | Lachnospiraceae_XPB1014_group | uncultured_bacterium* *Firmicutes | Bacilli | Lactobacillales | Streptococcaceae | Streptococcus*
	BCG_309	4.25	*Firmicutes | Clostridia | Oscillospirales | Ethanoligenenaceae | Incertae_Sedis | uncultured_bacterium* *Firmicutes | Clostridia | Christensenellales | Christensenellaceae | Christensenellaceae_R-7_group | uncultured_rumen*

^1^ The BCG markers were identified by linear discriminant analysis effect size (LEfSe) of BCG remaining in the reduced and optimized random forest classification model for prediction of forage type. Features were retained in the model based on feature importance scores [64,65,66]. ^2^ HAY-SP: initial standardized cool-season grass hay diet in the spring; HAY-FA: final standardized cool-season grass hay diet in the fall. ^3^ Amplicon sequence variants (ASV) were grouped into BCG using Sparse Cooccurrence Network Investigation for Compositional Data in Qiime 2 (v.2020.8) [40,62]. ^4^ For LEfSe, significance was set at LDA >2.0; *p* ≤ 0.05. ^5^ Taxonomic assignment of ASV was conducted using the most recent SILVA database (SSU 138).

**Table 4:** Bacterial co-abundance groups (BCG) identified as markers ^1^ of cool-season grass pasture.

Forage ^2^	BCG ^3^	LDA ^4^	ASV Taxonomic Lineage ^5^
**CSG-SP**	BCG_43	4.98	*Spirochaetota | Spirochaetia | Spirochaetales | Spirochaetaceae | Sphaerochaeta | uncultured_rumen* *Firmicutes | Negativicutes | Acidaminococcales | Acidaminococcaceae | Phascolarctobacterium* *Firmicutes | Clostridia | Oscillospirales | Oscillospiraceae | UCG-002 | uncultured_bacterium* *Firmicutes | Clostridia | Oscillospirales | UCG-010 | UCG-010 | uncultured_bacterium* *Firmicutes | Clostridia | Christensenellales | Christensenellaceae | Christensenellaceae_R-7_group*
	BCG_94	4.70	*Bacteroidota | Bacteroidia | Bacteroidales | Prevotellaceae | Prevotella | uncultured_Prevotellaceae* *Firmicutes | Clostridia | Clostridiales | Clostridiaceae | Sarcina | uncultured_bacterium* *Firmicutes | Clostridia | Clostridiales | Clostridiaceae | Clostridium_sensu_stricto_1*
	BCG_233	4.80	*Firmicutes | Clostridia | Clostridiales | Clostridiaceae | Clostridium_sensu_stricto_1* *Bacteroidota | Bacteroidia | Bacteroidales | Bacteroidales_BS11_gut_group | Bacteroidales_BS11_gut_group | uncultured_bacterium*
**CSG-FA**	BCG_45	4.35	*Firmicutes | Clostridia | Peptostreptococcales-Tissierellales | Anaerovoracaceae | Family_XIII_AD3011_group | uncultured_bacterium* *Firmicutes | Bacilli | Erysipelotrichales | Erysipelotrichaceae | Catenisphaera | uncultured_bacterium* *Actinobacteriota | Coriobacteriia | Coriobacteriales | Eggerthellaceae | Denitrobacterium | Denitrobacterium_detoxificans* *Firmicutes | Clostridia | Lachnospirales | Lachnospiraceae | Marvinbryantia | uncultured_rumen* *Firmicutes | Clostridia | Peptostreptococcales-Tissierellales | Anaerovoracaceae*
	BCG_48	4.79	*Firmicutes | Clostridia | Oscillospirales | [Eubacterium]_coprostanoligenes_group | [Eubacterium]_coprostanoligenes_group | uncultured_bacterium* *Bacteroidota | Bacteroidia | Bacteroidales | Bacteroidales_UCG-001 | Bacteroidales_UCG-001* *Bacteroidota | Bacteroidia | Bacteroidales | Rikenellaceae | Rikenellaceae_RC9_gut_group | uncultured_bacterium* *Firmicutes | Clostridia | Oscillospirales | Oscillospiraceae | NK4A214_group*
	BCG_51	4.36	*Firmicutes | Clostridia | Lachnospirales | Lachnospiraceae | Lachnospiraceae_XPB1014_group | uncultured_bacterium* *Firmicutes | Clostridia | Lachnospirales | Lachnospiraceae | Lachnospiraceae_UCG-009 | uncultured_bacterium* *Firmicutes | Clostridia | Peptostreptococcales-Tissierellales | Anaerovoracaceae | uncultured | uncultured_bacterium* *Firmicutes | Bacilli | Erysipelotrichales | Erysipelatoclostridiaceae | Erysipelatoclostridium | uncultured_bacterium*
	BCG_219	4.55	*Bacteroidota | Bacteroidia | Bacteroidales | Prevotellaceae | Alloprevotella | uncultured_bacterium* *Bacteroidota | Bacteroidia | Bacteroidales | Bacteroidales_RF16_group | Bacteroidales_RF16_group | uncultured_bacterium*
	BCG_275	4.45	*Firmicutes | Clostridia | Oscillospirales | Oscillospiraceae | NK4A214_group | uncultured_bacterium* *Verrucomicrobiota | Kiritimatiellae | WCHB1-41 | WCHB1-41 | WCHB1-41 | uncultured_bacterium*

^1^ The BCG markers were identified by linear discriminant analysis effect size (LEfSe) of BCG remaining in the reduced and optimized random forest classification model for prediction of forage type. Features were retained in the model based on feature importance scores [64,65,66]. ^2^ HAY-SP: initial standardized cool-season grass hay diet in the spring; HAY-FA: final standardized cool-season grass hay diet in the fall. ^3^ Amplicon sequence variants (ASV) were grouped into BCG using Sparse Cooccurrence Network Investigation for Compositional Data in Qiime 2 (v.2020.8) [40,62]. ^4^ For LEfSe, significance was set at LDA >2.0; *p* ≤ 0.05. ^5^ Taxonomic assignment of ASV was conducted using the most recent SILVA database (SSU 138).

**Table 5:** Bacterial co-abundance groups (BCG) identified as markers ^1^ of warm-season grass pasture.

Forage ^2^	BCG ^3^	LDA ^4^	ASV Taxonomic Lineage ^5^
**WSG**	BCG_35	4.68	*Verrucomicrobiota | Verrucomicrobiae | Verrucomicrobiales | Akkermansiaceae | Akkermansia | uncultured_bacterium* *Firmicutes | Clostridia | Christensenellales | Christensenellaceae | Christensenellaceae_R-7_group* *Fibrobacterota | Fibrobacteria | Fibrobacterales | Fibrobacteraceae | Fibrobacter | Fibrobacter_*sp. *Spirochaetota | Spirochaetia | Spirochaetales | Spirochaetaceae | Treponema | uncultured_bacterium* *Firmicutes | Clostridia | Peptostreptococcales-Tissierellales | Anaerovoracaceae | Family_XIII_AD3011_group | uncultured_bacterium*
	BCG_100	4.21	*Firmicutes | Clostridia | Oscillospirales | Oscillospiraceae | NK4A214_group* *Spirochaetota | Spirochaetia | Spirochaetales | Spirochaetaceae | Treponema* *Firmicutes | Clostridia | Oscillospirales | Oscillospiraceae | UCG-005 | metagenome*
	BCG_113	4.85	*Bacteroidota | Bacteroidia | Bacteroidales | Bacteroidales_RF16_group | Bacteroidales_RF16_group | uncultured_bacterium* *Firmicutes | Clostridia | Clostridiales | Clostridiaceae | Clostridium_sensu_stricto_1 | Clostridium_butyricum* *Firmicutes | Clostridia | Oscillospirales | Oscillospiraceae | Papillibacter | uncultured_bacterium*
	BCG_124	4.58	*Firmicutes | Clostridia | Christensenellales | Christensenellaceae | Christensenellaceae_R-7_group* *Synergistota | Synergistia | Synergistales | Synergistaceae | uncultured | uncultured_bacterium* *Firmicutes | Clostridia | Peptostreptococcales-Tissierellales | Anaerovoracaceae | Mogibacterium*
	BCG_259	4.46	*Firmicutes | Clostridia | Lachnospirales | Lachnospiraceae* *Firmicutes | Clostridia | Lachnospirales | Lachnospiraceae | [Eubacterium]_hallii_group | uncultured_bacterium*

^1^ The BCG markers were identified by linear discriminant analysis effect size (LEfSe) of BCG remaining in the reduced and optimized random forest classification model for prediction of forage type. Features were retained in the model based on feature importance scores [64,65]. ^2^ HAY-SP: initial standardized cool-season grass hay diet in the spring; HAY-FA: final standardized cool-season grass hay diet in the fall. ^3^ Amplicon sequence variants (ASV) were grouped into BCG using Sparse Cooccurrence Network Investigation for Compositional Data in Qiime 2 (v.2020.8) [40,62]. ^4^ For LEfSe, significance was set at LDA >2.0; *p* ≤ 0.05. ^5^ Taxonomic assignment of ASV was conducted using the most recent SILVA database (SSU 138).

**Table 6:** Fecal metabolite concentration ^1^ of horses adapted to hay diets and pasture forages.

Fecal Metabolites ^2^, ug g Feces ^−1^	Forage ^3^				
	HAY-SP	CSG-SP	WSG	CSG-FA	HAY-FA
**Total SCFA**	674 ± 76 ^ab^	1071 ± 121 ^c^	860 ± 98 ^ac^	1134 ± 129 ^c^	437 ± 50 ^b^
**Total BCFA**	48 ± 6 ^a^	136 ± 16 ^b^	113 ± 13 ^b^	126 ± 15 ^b^	70 ± 8 ^a^
**Acetate**	323 ± 34 ^ab^	473 ± 50 ^ac^	423 ± 45 ^ac^	524 ± 55 ^c^	229 ± 24 ^b^
**Butyrate**	101 ± 22 ^ab^	174 ± 30 ^a^	128 ± 22 ^a^	170 ± 29 ^a^	62 ± 11 ^b^
**Propionate**	217 ± 25 ^a^	365 ± 42 ^b^	274 ± 31 ^ab^	390 ± 45 ^b^	110 ± 13 ^c^
**Valerate**	12.0 ± 2.1 ^ab^	40.4 ± 7.2 ^c^	24.2 ± 4.3 ^bc^	35.3 ± 6.3 ^c^	18.1 ± 3.2 ^b^
**Hexanoate**	14.2 ± 3.3 ^a^	3.4 ± 0.8 ^b^	5.2 ± 1.2 ^b^	3.6 ± 0.8 ^b^	11.6 ± 2.7 ^a^
**Heptanoate**	2.8 ± 0.5 ^a^	0.8 ± 0.1 ^b^	1.2 ± 0.2 ^b^	0.8 ± 0.1 ^b^	1.7 ± 0.3 ^ab^
**Isobutyrate**	34.4 ± 3.5 ^a^	81.9 ± 8.3 ^b^	76.9 ± 7.8 ^b^	77.7 ± 7.9 ^b^	47.7 ± 4.8 ^a^
**Isovalerate**	13.5 ± 2.1 ^a^	51.2 ± 7.9 ^b^	35.0 ± 5.4 ^bc^	47.1 ± 7.3 ^b^	21.6 ± 3.4 ^ac^

^1^ Concentrations were determined by gas chromatography—mass spectroscopy (GC-MS). Data are presented as means ± SEM. ^2^ SCFA: short-chain fatty acids; BCFA: branched chain-fatty acids. ^3^ HAY-SP: initial standardized cool-season grass hay diet in the spring; CSG-SP: cool-season grass pasture in the spring; WSG: warm-season grass, either crabgrass or bermudagrass, in the summer slump period; CSG-FA: cool-season pasture in the fall; HAY-FA: final standardized cool-season grass hay diet in the fall. ^a,b,c^ Indicates significant differences between forages within rows (mixed model ANOVA with Tukey’s post hoc adjustment; *p* ≤ 0.05).

**Table 7:** Random forest regression accuracy ^1^ for predicting fecal variables and forage nutrients based on microbial composition.

	Model Accuracy ^2^			
	Full BCG Dataset ^2^		Reduced BCG Dataset ^3^	
	R^2^	*p*-Value	R^2^	*p*-Value
**Fecal Variables ^4^**				
Total SCFA	0.06	0.13	0.24	0.001
Total BCFA	0.08	0.07	0.28	0.0004
Acetate	0.05	0.19	0.29	0.0003
Butyrate	0.04	0.20	0.26	0.002
Propionate	0.03	0.31	0.17	0.008
Valerate	0.24	0.49	0.15	0.01
Hexanoate	0.26	0.0007	0.48	<0.0001
Heptanoate	0.11	0.03	0.24	0.001
Isobutyrate	0.01	0.54	0.27	0.0006
Isovalerate	0.26	0.0007	0.26	0.0007
Fecal pH	0.06	0.13	0.17	0.008
**Forage Nutrients**				
Digestible Energy	0.26	0.0008	0.34	<0.0001
Crude Protein	0.41	<0.0001	0.62	<0.0001
Acid Detergent Fiber	0.19	0.005	0.39	<0.0001
Neutral Detergent Fiber	0.27	0.0005	0.35	<0.0001
Non-Structural Carbohydrate	0.53	<0.0001	0.61	<0.0001
Water-Soluble Carbohydrate	0.52	<0.0001	0.67	<0.0001
Ethanol-Soluble Carbohydrate	0.10	0.04	0.18	0.006
Starch	0.21	0.003	0.40	<0.0001

^1^ Random forest regression modeling with nested cross-validation was conducted in Qiime2 (v.2020.8) [40,64]. ^2^ Initial model accuracies for random forest regressors were determined using the full feature set of bacterial co-abundance groups (BCG) and ungrouped amplicon sequence variants generated using Sparse Co-Occurrence Network Investigation for Compositional Data [62]. ^3^ Random forest regressors were then applied to a reduced BCG dataset (iterative reduction to 25 features most predictive of forage type [based on model importance scores]) [64,65]. ^4^ SCFA: short-chain fatty acids; BCFA: branched chain-fatty acids.

## Data Availability

The datasets generated and analyzed during the current study are available in the NCBI Sequence Read Archive at https://www.ncbi.nlm.nih.gov/sra (accessed on 31 July 2022), Bioproject: PRJNA864051, Accession numbers: SAMN30072246-SAMN30072285.

## Supplementary Materials

The following are available online at https://www.mdpi.com/article/10.3390/ani13050790/s1, Figure S1: Diagram of experimental design and sampling protocol, Figure S2: Insulinemic responses of horses to administration of oral sugar tests, Figure S3: Fasting plasma glucose and insulin prior to administration of oral sugar tests, Figure S4: Proxies for insulin sensitivity and the insulin secretory response in horses administered oral sugar tests, Table S1: Monthly average temperatures and precipitation totals and historical averages, Table S2: Horse condition measures following adaptation to hay diets and pasture forages, Table S3: Taxonomic classification of amplicon sequence variants (ASV) after quality filtering, chimera checking, and filtering low-abundance ASV.
