# Supplementary material for: Fecal Microbiota, Forage Nutrients, and Metabolic Responses of Horses Grazing Warm- and Cool-Season Grass Pastures
Source: Animals (Basel). 2023 Feb 22;13(5):790. doi: 10.3390/ani13050790 (PMC10000167; doi:10.3390/ani13050790)
Supplement: Supplementary file 1 [file animals-13-00790-s001.zip › Figure S1.pdf]

Figure S1. Diagram of experimental design and sampling protocol.

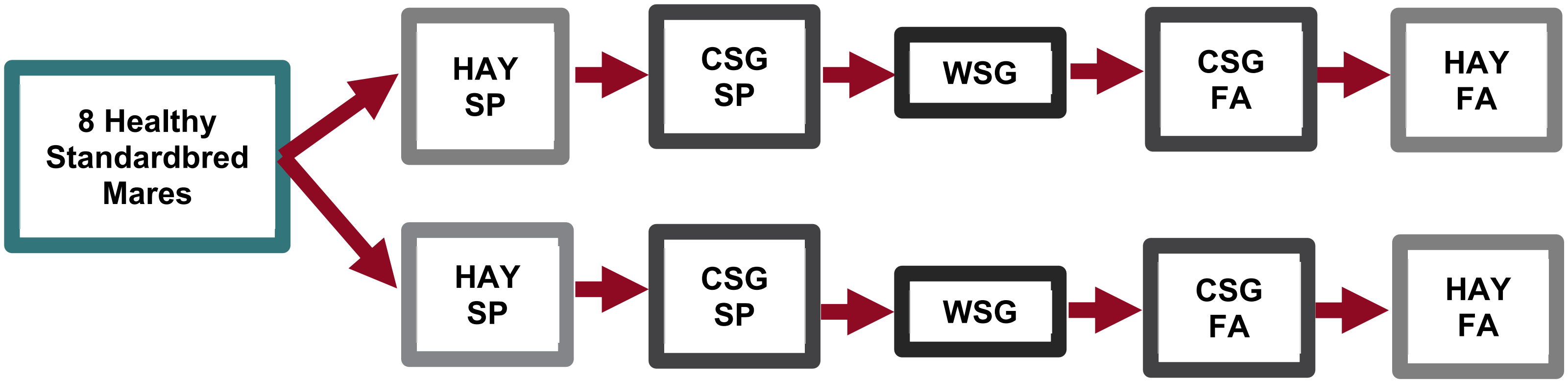

Eight healthy standardbred mares were randomly assigned to one of two grazing systems (n=4 per system). Fecal samples were collected after adaptation (17-21 d based on forage availability) to cool-season grass hay in the spring prior to pasture availability (HAY-SP), after grazing cool-season grass in the spring (CSG-SP), warm-season grass during the summer slump period (WSG), after a return to cool-season grass pasture in the fall (CSG-FA), and following a final standardized hay diet after the end of the grazing season (HAY-FA).
