# Supplementary material for: Fecal Microbiota, Forage Nutrients, and Metabolic Responses of Horses Grazing Warm- and Cool-Season Grass Pastures
Source: Animals (Basel). 2023 Feb 22;13(5):790. doi: 10.3390/ani13050790 (PMC10000167; doi:10.3390/ani13050790)
Supplement: Supplementary file 1 [file animals-13-00790-s001.zip › Figure S2.pdf]

Figure S1. Insulinemic responses of horses to administration of oral sugar tests (OST).

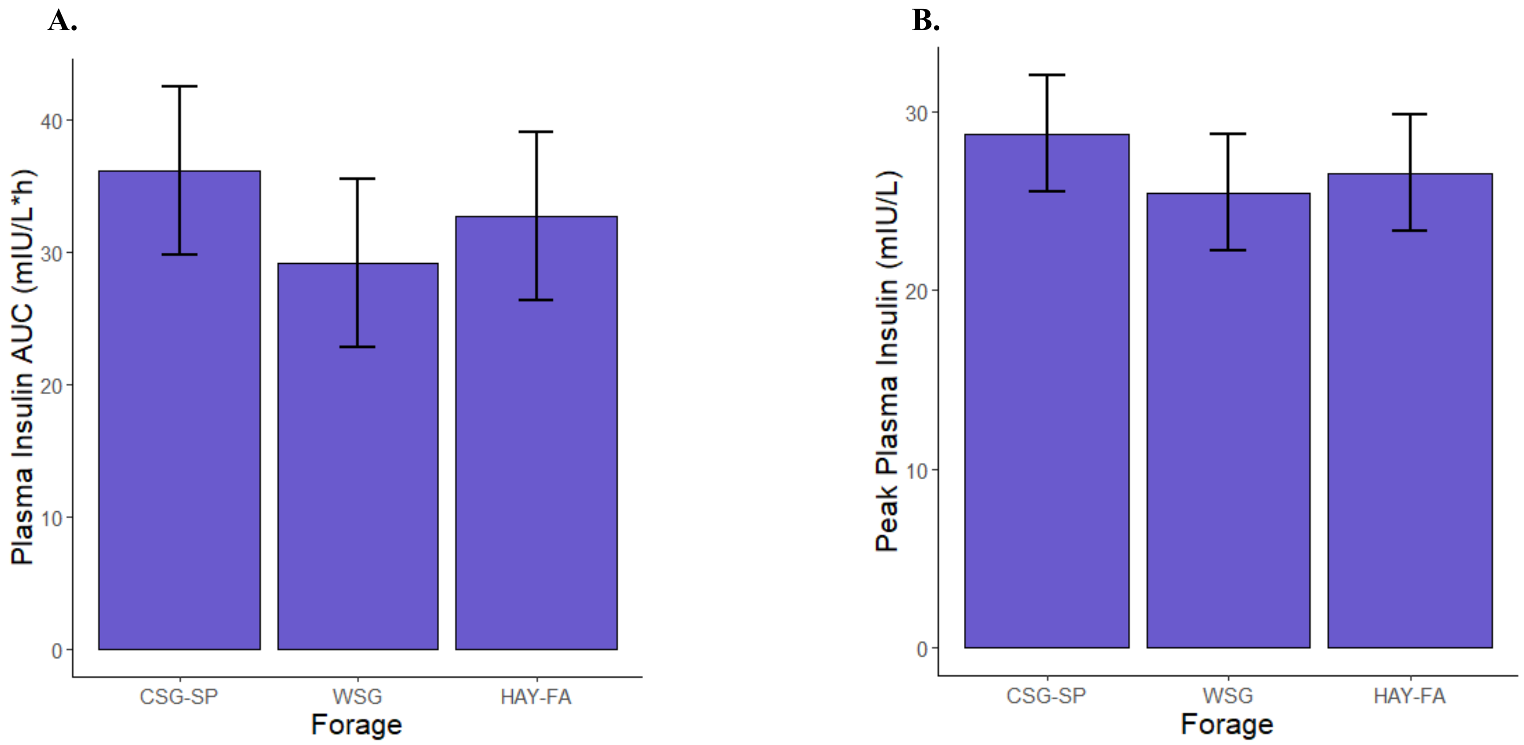

The OST were administered to horses adapted to cool-season grass pasture in the spring (CSG-SP) and warm-season grass in the summer slump period (WSG) within an integrated rotational grazing system in addition to a standardized cool-season grass hay diet in the fall at the end of the grazing season (HAY-FA). Insulinemic responses analyzed included the positive incremental area under the curve (AUC) (a) and peak plasma glucose (b). There were no treatment differences in insulinemic responses (mixed model ANOVA with Tukey's post hoc adjustment; $P > 0.05$). Data are presented as means ± SEM.
