# Supplementary material for: Fecal Microbiota, Forage Nutrients, and Metabolic Responses of Horses Grazing Warm- and Cool-Season Grass Pastures
Source: Animals (Basel). 2023 Feb 22;13(5):790. doi: 10.3390/ani13050790 (PMC10000167; doi:10.3390/ani13050790)
Supplement: Supplementary file 1 [file animals-13-00790-s001.zip › Figure S3.pdf]

Figure S2. Fasting plasma glucose and insulin prior to administration of oral sugar tests (OST).

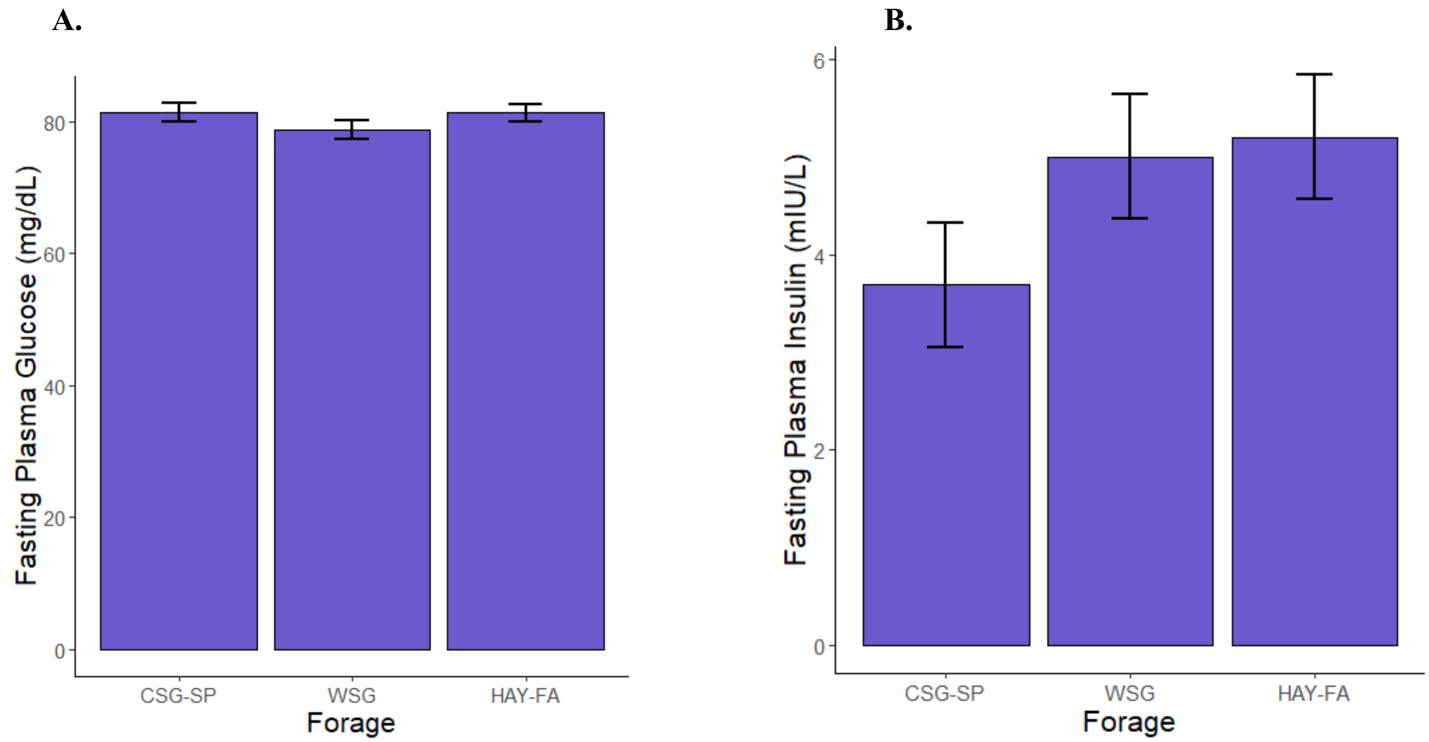

Fasting plasma glucose (a) and insuin (b) prior to administration of oral sugar tests conducted in horses adapted to cool-season grass pasture in the spring (CSG-SP) and warm-season grass in the summer slump period (WSG) within an integrated rotational grazing system in addition to a standardized cool-season grass hay diet in the fall at the end of the grazing season (HAY-FA). Fasting glucose and insulin concentrations represent the mean of two consecutive fasting baseline samples were collected at a 15-min interval prior to administration of the OST. Neither fasting glucose or insulin differed by treatment (mixed model ANOVA with Tukey's post hoc adjustment; $P > 0.05$). Data are presented as means ± SEM.
