# Supplementary material for: Fecal Microbiota, Forage Nutrients, and Metabolic Responses of Horses Grazing Warm- and Cool-Season Grass Pastures
Source: Animals (Basel). 2023 Feb 22;13(5):790. doi: 10.3390/ani13050790 (PMC10000167; doi:10.3390/ani13050790)
Supplement: Supplementary file 1 [file animals-13-00790-s001.zip › Figure S4.pdf]

Figure S3. Proxies for insulin sensitivity and the insulin secretory response in horses administered oral sugar tests (OST).

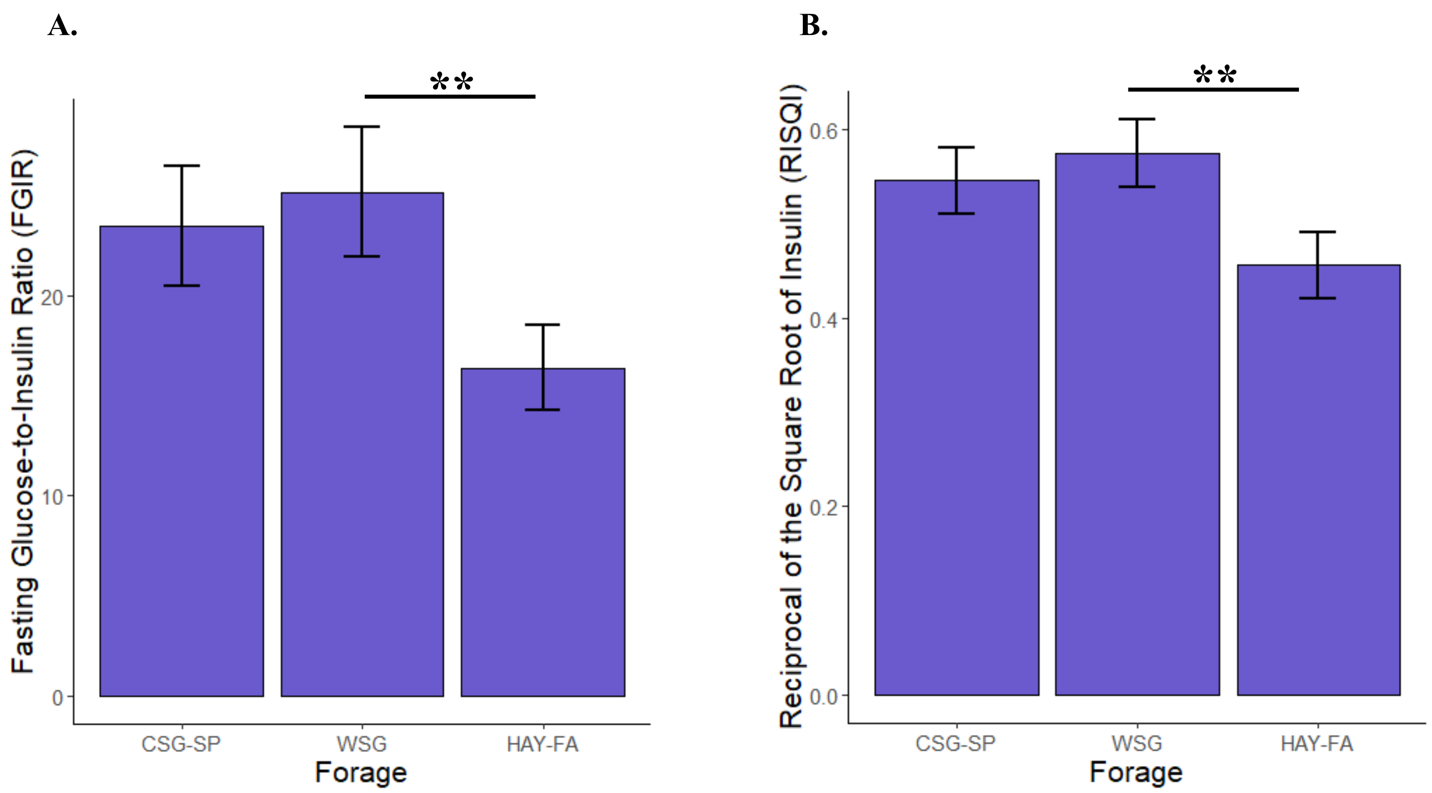

C.

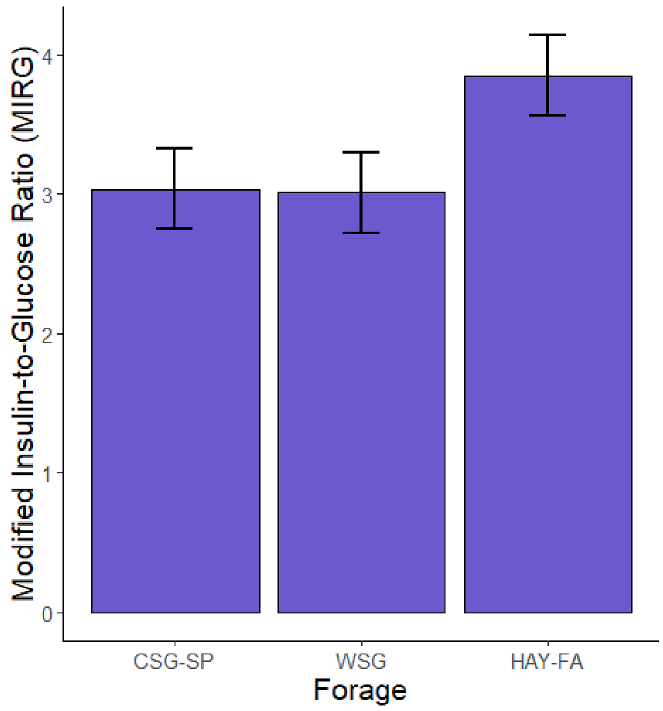

The OST were administered to horses adapted to cool-season grass pasture in the spring (CSG-SP) and warm-season grass in the summer slump period (WSG) within an integrated rotational grazing system in addition to a standardized cool-season grass hay diet in the fall at the end of the grazing season (HAY-FA). Proxies analyzed included the fasting glucose-to-insulin ratio (FGIR; a), the reciprocal of the square root of insulin (RISQI; b), and the modified insulin-to-glucose ratio (MIRG; c). Data are presented as means ± SEM. A double asterisk indicates a trend for differences between forages at $P \leq 0.10$ (mixed model ANOVA with Tukey's post hoc adjustment).
