# Supplementary material for: Fecal Microbiota, Forage Nutrients, and Metabolic Responses of Horses Grazing Warm- and Cool-Season Grass Pastures
Source: Animals (Basel). 2023 Feb 22;13(5):790. doi: 10.3390/ani13050790 (PMC10000167; doi:10.3390/ani13050790)
Supplement: Supplementary file 1 [file animals-13-00790-s001.zip › Table S1.pdf]

Table S1. Weather data[1].

Month	Average Temperature, °C		Total Precipitation, cm	
	2018	Historical Average	2018	Historical Average
**May**	18.22	16.17	14.86	10.21
**Jun**	21.17	21.00	12.60	9.93
**Jul**	24.61	23.78	10.92	12.32
**Aug**	25.22	22.78	10.62	11.89
**Sep**	21.39	19.17	22.40	10.29
**Oct**	13.44	12.94	8.13	8.97
**Nov**	5.83	7.11	22.15	8.81
**Dec**	3.28	1.39	14.76	9.58

[1] Weather data were obtained for the New Brunswick Station through the Office of the New Jersey State Climatologist website [81].
