# Supplementary material for: Fecal Microbiota, Forage Nutrients, and Metabolic Responses of Horses Grazing Warm- and Cool-Season Grass Pastures
Source: Animals (Basel). 2023 Feb 22;13(5):790. doi: 10.3390/ani13050790 (PMC10000167; doi:10.3390/ani13050790)
Supplement: Supplementary file 1 [file animals-13-00790-s001.zip › Table S2.pdf]

Table S2. Horse condition measures following adaptation to hay diets and pasture forages.

Horse Condition Measure	Forage[1]					SEM
	HAY-SP	CSG-SP	WSG	CSG-FA	HAY-FA	
**Body Weight, kg**	536 [a]	551 [ab]	553 [b]	541 [ab]	531 [a]	18
**Body Condition Score, scale: 1-9** [2]	5.44	5.62	5.62	5.38	5.12	0.26

[1] HAY-SP: initial standardized cool-season grass hay diet in the spring; CSG-SP: cool-season grass pasture in the spring; WSG: warm-season grass, either crabgrass or bermudagrass, in the summer slump period; CSG-FA: cool-season pasture in the fall; HAY-FA: final standardized cool-season grass hay diet in the fall.

[2] Body condition score was evaluated using the Henneke Body Condition Score scale, with scores assigned on a scale of 1-9 (1 = emaciated and 9 = morbidly obese) [83].

[a,b] Indicates significant difference within rows (mixed model ANOVA with Tukey's post hoc adjustment; $P \leq 0.05$)
