# Supplementary material for: Fecal Microbiota, Forage Nutrients, and Metabolic Responses of Horses Grazing Warm- and Cool-Season Grass Pastures
Source: Animals (Basel). 2023 Feb 22;13(5):790. doi: 10.3390/ani13050790 (PMC10000167; doi:10.3390/ani13050790)
Supplement: Supplementary file 1 [file animals-13-00790-s001.zip › Table S3.pdf]

Table S3. Taxonomic classification[1] of all amplicon sequence variants (ASV)[2].

Taxonomic Lineage							ASV Count[3]
**Kingdom**	**Phylum**	**Class**	**Order**	**Family**	**Genus**	**Species**	
Archaea	*Euryarchaeota*	*Methanobacteria*	*Methanobacteriales*	*Methanobacteriaceae*	*Methanobrevibacter*	-------------	*5*
	Halobacterota	*Methanomicrobia*	*Methanomicrobiales*	*Methanocorpusculaceae*	*Methanobrevibacter*	*uncultured archeon*	*6*
	Thermoplasmatota	*Thermoplasmata*	*Methanomassiliicoccales*	*Methanomethylophilaceae*	*uncultured*	-------------	*1*
Bacteria	-------------	-------------	-------------	-------------	-------------	-------------	*5*
	Actinobacteriota	*Coriobacteriia*	*Coriobacteriales*	*Coriobacteriales Incertae Sedis*	*Phoenicibacter*	*uncultured bacterium*	*4*
					uncultured	*Uncultured bacterium*	*1*
				Eggerthellaceae	-------------	-------------	*2*
					Denitrobacterium	*D. detoxificans*	*1*
					Parvibacter	*uncultured bacterium*	*1*
					uncultured	*uncultured bacterium*	*3*
				uncultured	*uncultured*	*uncultured bacterium*	*1*
Bacteria	*Armatimonadota*	-------------	-------------	-------------	-------------	-------------	*1*
		uncultured	*uncultured*	*uncultured*	-------------	-------------	*1*
					uncultured	*uncultured bacterium*	*4*
Bacteria	*Bacteroidetes*	*Bacteroidia*	-------------	-------------	-------------	-------------	*4*
			Bacteroidales	-------------	-------------	-------------	*9*
				Bacteroidales BS11 gut group	*Bacteroidales BS11 gut group*	-------------	*1*
						uncultured bacterium	*4*
				Bacteroidales RF16 group	*Bacteroidales RF16 group*	-------------	*1*
						uncultured bacterium	*12*
				Bacteroidales UCG-001	*Bacteroidales UCG-001*	-------------	*4*
						uncultured bacterium	*5*
						uncultured rumen	*4*
				Bacteroidetes BD2-2	*Bacteroidetes BD2-2*	*uncultured rumen*	*1*

CAP-aah99b04	*CAP-aah99b04*	*uncultured bacterium*	*1*
Dysgonomonadaceae	*Uncultured*	*uncultured bacterium*	*1*
F082	*F082*	-------------	*2*
		Bacteriodales bacterium	*1*
		uncultured bacterium	*32*
gir-aah93h0	*gir-aah93h0*	*uncultured bacterium*	*1*
M2PB4-65 termite group	*M2PB4-65 termite group*	*uncultured bacterium*	*4*
Marinifilaceae	*uncultured*	*uncultured bacterium*	*2*
Muribaculaceae	*Muribaculaceae*	-------------	*1*
		uncultured bacterium	*3*
p-251-o5	*p-251-o5*	*uncultured bacterium*	*28*
Paludibacteraceae	*uncultured*	*uncultured bacterium*	*9*
Prevotellaceae	-------------	-------------	*1*
	Alloprevotella	-------------	*7*
		uncultured bacterium	*4*
	Prevotella	-------------	*10*
		uncultured Prevotellaceae	*1*
	Prevotellaceae Ga6A1 group	*unidentified rumen*	*1*
	Prevotellaceae UCG-001	*uncultured bacterium*	*7*
		uncultured Prevotellaceae	*2*
		wallaby gut	*6*
	Prevotellaceae UCG-003	-------------	*3*
	Prevotellaceae UCG-004	*uncultured bacterium*	*12*
Rikenellaceae	-------------	-------------	*1*
	Alistipes	*uncultured bacterium*	*1*
	dgA-11 gut group	*uncultured bacterium*	*2*
	hoa5-07d05 gut group	*uncultured bacterium*	*7*
	Mucinivorans	*uncultured bacterium*	*1*

					Rikenellaceae RC9 gut group	-------------	*19*
						uncultured bacterium	*79*
						uncultured Bacteriodales	*2*
						uncultured beta	*6*
						uncultured Rikenella	*1*
						wallaby gut	*2*
					SP3-e08	*uncultured bacterium*	*15*
				uncultured	*uncultured*	*uncultured bacterium*	*2*
Bacteria	*Chloroflexi*	*Anaerolineae*	*Anaerolineales*	*Anaerolineaceae*	*Flexilinea*	*uncultured bacterium*	*2*
Bacteria	*Cyanobacteria*	*Vampirivibrionia*	*Gastranaerophilales*	*Gastranaerophilales*	*Gastranaerophilales*	-------------	*1*
					Gastranaerophilales	*uncultured bacterium*	*1*
Bacteria	*Desulfobacterota*	*Desulfovibrionia*	*Desulfovibrionales*	*Desulfovibrionaceae*	*Desulfovibrio*	-------------	*3*
					Mailhella	-------------	*1*
						uncultured bacterium	*1*
Bacteria	*Elusimicrobiota*	*Endomicrobia*	*Endomicrobales*	*Endomicrobiaceae*	*Endomicrobium*	*uncultured bacterium*	*1*
Bacteria	*Fibrobacterota*	*Fibrobacteria*	*Fibrobacterales*	*Fibrobacteraceae*	*Fibrobacter*	-------------	*1*
						Fibrobacter sp	*3*
Bacteria	*Firmicutes*	-------------	-------------	-------------	-------------	-------------	*2*
		Bacilli	*Bacillales*	*Bacillaceae*	*Bacillus*	-------------	*1*
			Erysipelotrichales	*Erysipelatoclostridiaceae*	-------------	-------------	*2*
					Candidatus Stoquefichus	*Candidatus Stoquefichus*	*1*
					Erysipelatoclostridium	*uncultured bacterium*	*2*
					UCG-004	-------------	*2*
						uncultured bacterium	*7*
					uncultured	*Uncultured Erysipelotrichaceae*	*1*
					[Anaerorhabdus] furcosa group	*uncultured rumen*	*1*
					Catenisphaera	*uncultured bacterium*	*4*
					uncultured	*uncultured bacterium*	*3*

	Izemoplasmatales	*Izemoplasmatales*	*Izemoplasmatales*	-------------	*1*
				uncultured bacterium	*3*
	Lactobacillales	*Lactobacillaceae*	*Lactobacillus*	-------------	*1*
				L. equigenerosi	*1*
				uncultured methanogenic	*2*
			Streptococcus	-------------	*1*
	Paenibacillales	*Paenibacillaceae*	*Paenibacillus*	-------------	*1*
				P. turicensis	*1*
	RF39	*RF39*	*RF39*	-------------	*12*
				uncultured bacterium	*1*
				uncultured prokaryote	*1*
Clostridia	-------------	-------------	-------------	-------------	*15*
	Caldicoprobacterales	*Caldicoprobacteraceae*	*Caldicoprobacter*	*uncultured bacterium*	*1*
	Christensenellales	*Christensenellaceae*	*Christensenellaceae R-7 group*	-------------	*78*
			-------------	-------------	*1*
				Christensenella sp.	*2*
				gut metagenome	*1*
				human gut	*1*
				uncultured bacterium	*5*
				uncultured Clostridia	*2*
				uncultured prokaryote	*2*
				uncultured rumen	*12*
				unidentified rumen	*3*
	Clostridia	*Hungateiclostridiaceae*	*Ruminiclostridium*	*unculture rumen*	*2*
			Saccharofermentans	-------------	*5*
			Saccharofermentans	*Clostridiales bacterium*	*1*
				Ruminococcaceae bacterium	*1*
				uncultured bacterium	*4*
			uncultured	*uncultured bacterium*	*1*
	Clostridia UCG-014	*Clostridia UCG-014*	*Clostridia UCG-014*	-------------	*12*
				uncultured bacterium	*4*

Clostridia vadinBB60 group	*Clostridia vadinBB60 group*	*Clostridia vadinBB60 group*	-------------	*2*
			Clostridiales bacterium	*1*
			metagenome	*1*
			uncultured bacterium	*5*
Clostridiales	*Clostridiaceae*	*Clostridium sensu stricto 1*	-------------	*4*
			Clostridium butyricum	*2*
		Sarcina	*uncultured bacterium*	*1*
Eubacteriales	*Anaerofustaceae*	*Anaerofustis*	*uncultured bacterium*	*1*
	Eubacteriaceae	*Eubacterium*	-------------	*3*
			uncultured bacterium	*3*
Lachnospirales	-------------	-------------	-------------	*3*
	Defluviitaleaceae	*Defluviitaleaceae UCG-011*	*uncultured bacterium*	*2*
	Lachnospiraceae	-------------	-------------	*74*
		[Eubacterium] hallii group	*uncultured bacterium*	*8*
		Acetitomaculum	*uncultured bacterium*	*3*
		Agathobacter	*uncultured rumen*	*1*
		Blautia	-------------	*6*
		Cellulosilyticum	*uncultured bacterium*	*3*
			uncultured Clostridia	*1*
		Coprococcus	-------------	*2*
			uncultured rumen	*1*
		FD2005	*uncultured bacterium*	*1*
		Frisingicoccus	*uncultured bacterium*	*1*
		Johnsonella	*Uncultured Lachnospiraceae*	*1*
		Lachnoclostridium	-------------	*2*
			uncultured bacterium	*1*
		Lachnospiraceae AC2044 group	-------------	*3*
			uncultured bacterium	*7*
			uncultured rumen	*3*

Lachnospiraceae FCS020 group	*uncultured bacterium*	*1*
Lachnospiraceae ND3007 group	*uncultured Lachnospiraceae*	*3*
	uncultured rumen	*2*
Lachnospiraceae NK4A136 group	-------------	*2*
	uncultured rumen	*2*
Lachnospiraceae NK4B4 group	*uncultured bacterium*	*2*
Lachnospiraceae UCG-002	-------------	*1*
Lachnospiraceae UCG-006	*uncultured rumen*	*1*
Lachnospiraceae UCG-008	*uncultured Lachnospiraceae*	*1*
	uncultured rumen	*1*
Lachnospiraceae UCG-009	*uncultured bacterium*	*9*
Lachnospiraceae UCG-010	*uncultured bacterium*	*1*
	uncultured rumen	*1*
Lachnospiraceae XPB1014 group	-------------	*4*
	uncultured bacterium	*9*
Marvinbryantia	-------------	*5*
	uncultured bacterium	*5*
	uncultured Lachnospiraceae	*1*
	uncultured rumen	*1*
Oribacterium	*uncultured bacterium*	*4*
Probable genus 10	*uncultured bacterium*	*1*
Pseudobutyrivibrio	-------------	*1*
Roseburia	-------------	*2*
Syntrophococcus	*uncultured bacterium*	*1*
	uncultured rumen	*2*
uncultured	-------------	*1*

Monoglobales	*Monoglobaceae*	*Monoglobus*	-------------	*1*
			uncultured bacterium	*7*
			uncultured Clostridiaceae	*1*
			uncultured rumen	*1*
Oscillospirales	-------------	-------------	-------------	*10*
	[Eubacterium] coprostanoligenes group	*[Eubacterium] coprostanoligenes group*	-------------	*21*
			uncultured bacterium	*15*
			uncultured rumen	*5*
			uncultured Ruminococcaceae	*2*
	Butyricicoccaceae	*UCG-009*	*uncultured bacterium*	*3*
	Ethanoligenenaceae	*Incertae Sedis*	*uncultured bacterium*	*1*
	Oscillospiraceae	-------------	-------------	*20*
		Colidextribacter	-------------	*1*
			uncultured rumen	*2*
		NK4A214 group	-------------	*25*
			uncultured bacterium	*5*
			uncultured rumen	*10*
			uncultured Ruminococcaceae	*1*
		Papillibacter	*uncultured bacterium*	*2*
			uncultured Clostridiaceae	*2*
			uncultured Clostridiales	*1*
		UCG-002	-------------	*13*
			uncultured bacterium	*4*
		UCG-005	-------------	*9*
			Metagenome	*1*
			uncultured bacterium	*2*
			uncultured organism	*2*
			uncultured rumen	*7*

		UCG-007	uncultured rumen	2
		uncultured	-------------	2
		uncultured	uncultured	1
		V9D2013 group	-------------	1
			uncultured bacterium	1
	Ruminococcaceae	-------------	-------------	4
		Candidatus Soleaferrea	-------------	1
			Uncultured Acetanaerobacterium	1
			uncultured bacterium	2
		Caproiciproducens	-------------	3
		Fournierella	Uncultured Firmicutes	1
		Pygmaiobacter	uncultured bacterium	3
		Ruminococcus	-------------	10
			Bacterium YRD2003	1
			R. flavefaciens	3
			Ruminococcus sp.	1
			uncultured Clostridiaceae	1
		Subdoligranulum	uncultured Ruminococcaceae	1
		uncultured	-------------	7
			uncultured bacterium	7
			uncultured Clostridiales	1
			uncultured rumen	2
	UCG-010	UCG-010	-------------	53
			uncultured bacterium	32
			uncultured Clostridia	1
			uncultured organism	1
			uncultured Ruminococcaceae	2
			Unidentified	1
	UCG-011	UCG-011	uncultured rumen	2

			Peptococcales	*Peptococcaceae*	-------------	-------------	*1*
					uncultured	-------------	*2*
						uncultured bacterium	*1*
						unidentified	*1*
				Anaerovoracaceae	-------------	-------------	*6*
					[Eubacterium] brachy group	*Uncultured bacterium*	*1*
					[Eubacterium] nodatum group	-------------	*1*
					Anaerovorax	-------------	*3*
						uncultured bacterium	*1*
						uncultured rumen	*6*
					Family XIII AD3011 group	-------------	*1*
						uncultured bacterium	*7*
					Family XIII UCG-001	*uncultured bacterium*	*1*
						uncultured rumen	*2*
					Mogibacterium	-------------	*1*
						uncultured bacterium	*1*
					uncultured	*uncultured bacterium*	*1*
			Peptostreptococcales -Tissierellales	-------------	-------------	-------------	*1*
		Negativicutes	*Acidaminococcales*	*Acidaminococcaceae*	*Phascolarctobacterium*	-------------	*6*
						uncultured bacterium	*4*
			Veillonellales-Selenomonadales	*Selenomonadaceae*	-------------	-------------	*1*
					Anaerovibrio	*uncultured bacterium*	*1*
					Quinella	-------------	*1*
					Schwartzia	-------------	*1*
				Veillonellaceae	*Veillonella*	*V. caviae*	*1*
		Syntrophomonadia	*Syntrophomonadales*	*Syntrophomonadaceae*	-------------	-------------	*1*
		uncultured	*uncultured*	*uncultured*	*uncultured*	*iron-reducing bacterium*	*1*
Bacteria	*Planctomycetota*	*Planctomycetes*	*Pirellulales*	*Pirellulaceae*	*p-1088-a5 gut group*	*uncultured bacterium*	*5*

		vadinHA49	*vadinHA49*	*vadinHA49*	*vadinHA49*	-------------	*1*
						uncultured bacterium	*1*
Bacteria	*Proteobacteria*	*Alphaproteobacteria*	*Rhodospirillales*	-------------	-------------	-------------	*1*
				uncultured	*uncultured*	-------------	*2*
						gut metagenome	*1*
						Uncultured rumen	*4*
		Gammaproteobacteria	*Burkholderiales*	-------------	-------------	-------------	*1*
				T34	*T34*	*uncultured bacterium*	*1*
Bacteria	*Spirochaetota*	*MVP-15*	*MVP-15*	*MVP-15*	*MVP-15*	-------------	*2*
						uncultured bacterium	*1*
		Spirochaetia	*Spirochaetales*	*Spirochaetaceae*	*Sediminispirochaeta*	-------------	*2*
					Sphaerochaeta	-------------	*1*
						uncultured bacterium	*3*
						uncultured rumen	*7*
					Treponema	-------------	*5*
						T. ruminis	*1*
						uncultured bacterium	*24*
Bacteria	*Synergistota*	*Synergistia*	*Synergistales*	*Synergistaceae*	-------------	-------------	*1*
					Cloacibacillus	*uncultured bacterium*	*1*
					uncultured	*uncultured bacterium*	*1*
Bacteria	*Verrucomicrobiota*	*Kiritimatiellae*	*WCHB1-41*	*WCHB1-41*	*WCHB1-41*	-------------	*6*
						uncultured bacterium	*21*
		Lentisphaeria	*Victivallales*	*vadinBE97*	*vadinBE97*	-------------	*1*
		Verrucomicrobiae	*LD1-PB3*	*LD1-PB3*	*LD1-PB3*	*uncultured bacterium*	*1*
			Pedosphaerales	*Pedosphaeraceae*	*DEV114*	-------------	*1*
						uncultured bacterium	*1*
			Verrucomicrobiales	*Akkermansiaceae*	*Akkermansia*	*uncultured bacterium*	*4*

[1] Taxonomic assignment was conducted in Qiime 2 using the most recent SILVA database (SSU 138).
[2] The ASV represent those retained after quality filtering, chimera checking, and filtering low abundance, but prior to rarefaction for subsequent diversity analyses.
[3] Counts of individual ASV with a shared lineage.
